# Supplementary material for: Discovering functionally important sites in proteins
Source: Nat Commun. 2023 Jul 13;14:4175. doi: 10.1038/s41467-023-39909-0 (PMC10345196; doi:10.1038/s41467-023-39909-0)
Supplement: Supplementary file 1 — Supplementary Information [file 41467_2023_39909_MOESM1_ESM.pdf]

Discovering functionally important sites in proteins: supplementary information

Matteo Cagiada[1], Sandro Bottaro[1], Søren Lindemose[1], Signe M. Schenstrøm[1], Amelie Stein[1], Rasmus Hartmann-Petersen[1], and Kresten Lindorff-Larsen[1]

[1] *Linderstrøm-Lang Centre for Protein Science, Department of Biology, University of Copenhagen, DK-2200 Copenhagen, Denmark*

1. FIGURES

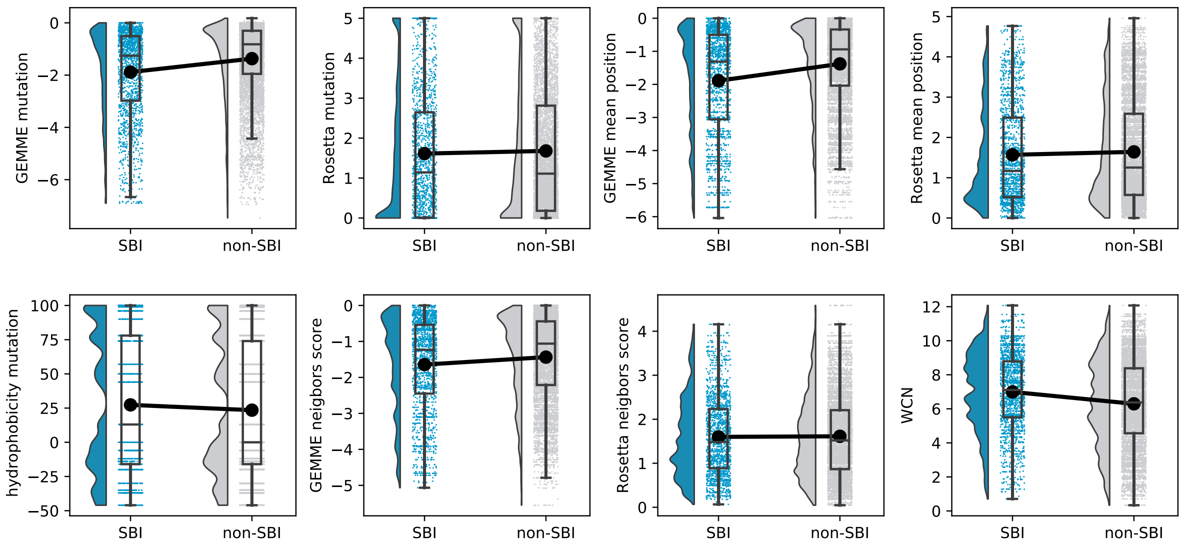

Supplementary Figure 1. Comparing values of the individual features for SBI and non-SBI variants. Using data from the training set, consisting of 9945 individual variants, each raincloud plot shows, for each feature used in the model, the distribution of feature values in the training proteins for variants belonging to the SBI class (as assigned by experiments) or in one of the other three classes (WT-like, total loss and 'low abundance, high activity'). Each plot also shows the data statistics with a boxplot, where the central line represents the median values, the boundaries of the box represent the first quartile (Q1; bottom) and the third quartile (Q3; top), and the boundaries of the whiskers are evaluated by summing to the nearest quartile 1.5 times the inter-quartile range, defined as Q3-Q1. The comparison between the medians is shown by a black line connecting the two medians.. Raw data are also displayed as points under the box plot.

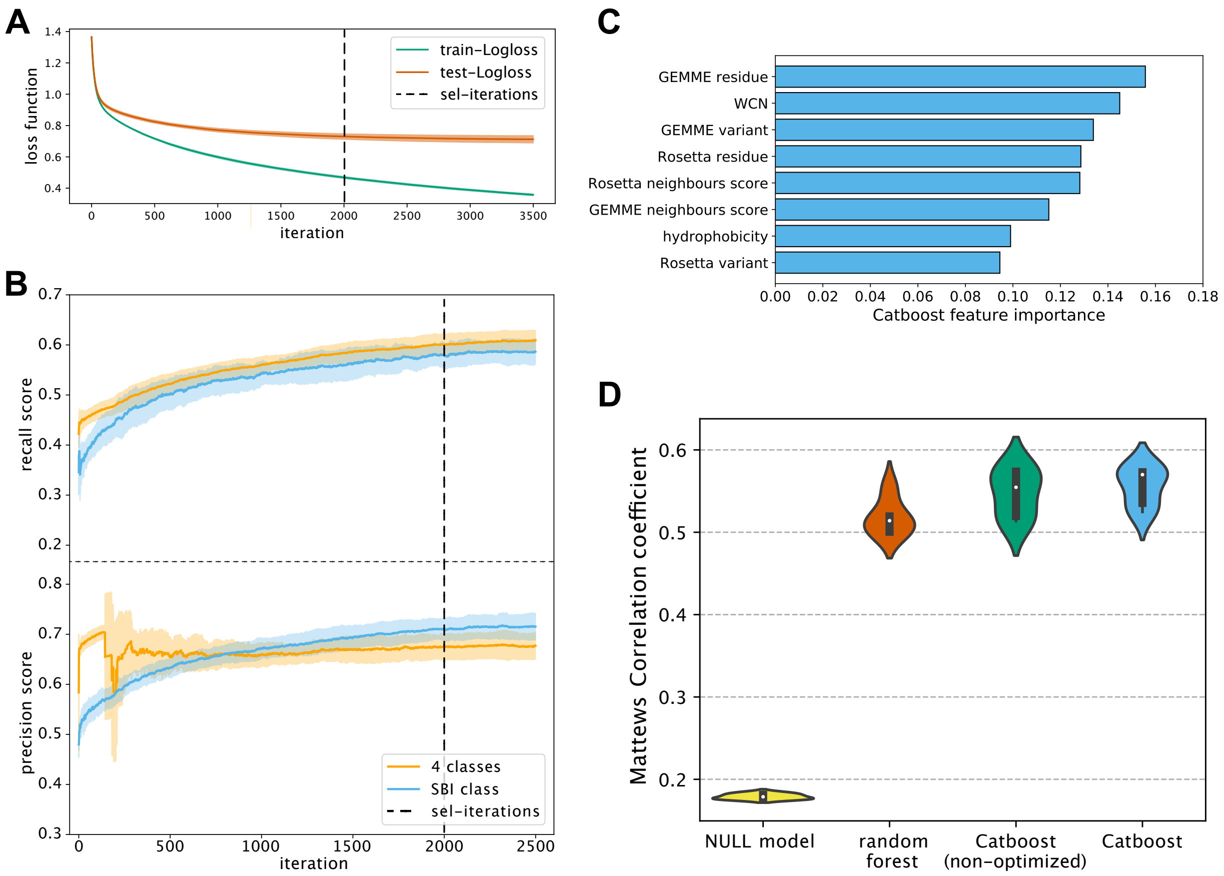

Supplementary Figure 2. Details of the variant classifier training process. (A) Changes in the loss function, obtained using a 5-fold cross validation protocol, for the (green) training data and (red) test data during model training. The solid lines represent the median values and the coloured areas report the standard deviation. (B) Progress of performance (measuring recall and precision on the test set of a 5-fold cross validation procedure) of the Catboost classifier during training. The yellow lines represent the median recall and precision for correctly classifing the variant in one of the four classes, while the blue lines show the median performance of the model for classifing the SBI variants. The shaded areas in panels A and B represent the standard deviation and the selected number of iterations is shown as a black vertical line. (C) Feature importance for each of the features used to train the model. (D) Comparison of the performance, on the test set (with 1989 variant tested) of a 5-fold cross validation procedure, of the Catboost model (using the Matthews' correlation coefficient) a Catboost version before optimizing hyper parameters, an optimized Random Forest model, and a Null model (which always returns the most frequent class label). The white points inside the 'violin plots' represent the median values and the black squared areas represent one standard deviation.

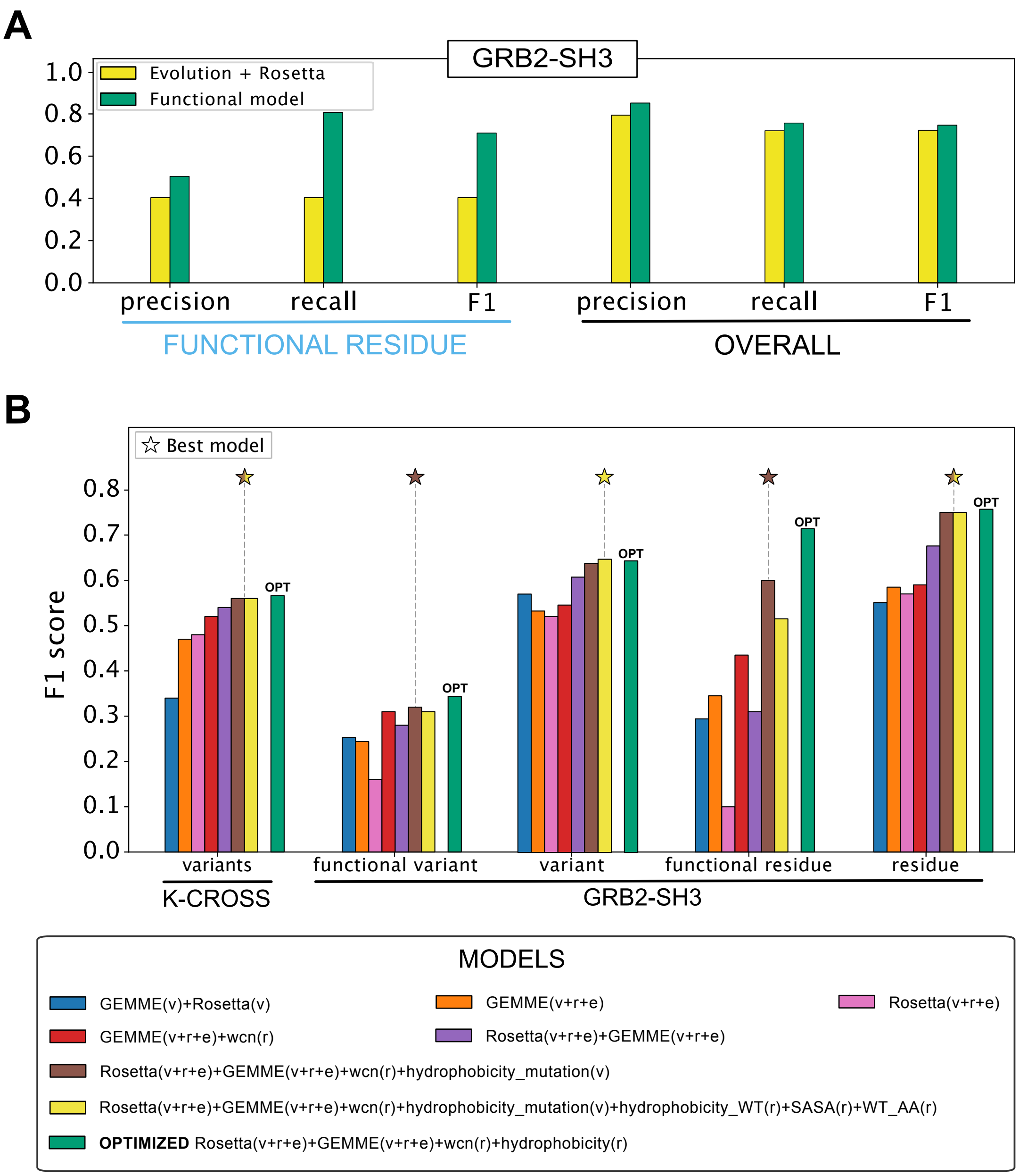

Supplementary Figure 3. Benchmarks of our functional sites model. (A) Comparison of the predictions of residue classes in the GRB2 SH3 domain using either our optimized model (green) or simply using cutoff values for evolutionary conservation and thermodynamic stability changes (yellow). The three leftmost bars show the results for the subset of functional residues, while the three rightmost series report the results for all the positions predicted. In both the cases precision, recall and F1-score are used as metrics. (B) Comparison between results from our vanilla model (in brown) with vanilla models trained using other sets of features. Results from our final version with optimized hyperparameters are reported (in green). F1 score is shown on the y-axis and the stars highlight the best model for each set (excluding the fully optimized model). The leftmost set reports the results on the test set (with 1989 variant tested) obtained from a 5-fold cross validation procedure, while the other sets show the scores for GRB2-SH3 domain, both for the functional residue subset (64 variants and 5 residues) and for all the residues (1053 variants and 56 residues). The legend reports which features were used with v, r and e representing variants, residues and environment, respectively (see Methods for list of features).

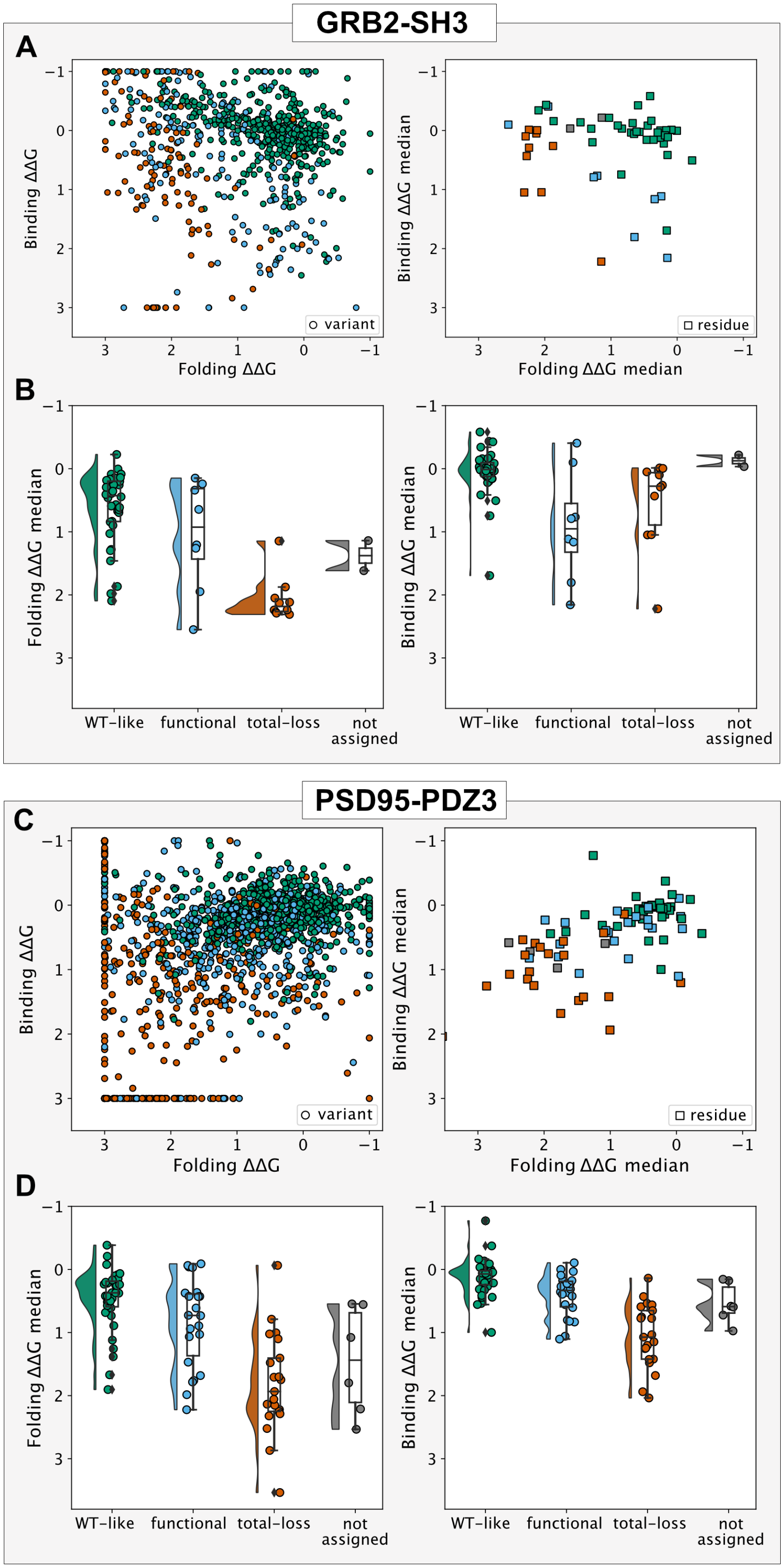

Supplementary Figure 4. Comparison of experiments and predictions for the GRB2-SH3 and PSD95-PDZ3 domains. (A, C) For each of the two domains we used our model to classify variants and residues and compared to $\Delta\Delta G$ values for domain stability (Folding $\Delta\Delta G$) and peptide binding (Binding $\Delta\Delta G$) inferred from experiments. Variants and residues are coloured according to the predicted class (WT-like: green; SBI/Functional site: blue; Total-loss: red; Not assigned: grey). (B, D) Rain-cloud plots of the median value of the folding or stability $\Delta\Delta G$ values for different positions divided into the different classifications. The box plots show the median and quartiles (of the residue median values). For GRB2-SH3 (A,B) we analysed a total of 1053 variants and 56 residues, whereas for PSD95-PDZ3 (C,D) we analysed a total of 1498 variants and 84 residues. In each boxplot, the central line represents the median values, the boundaries of the box represent the first quartile (Q1; bottom) and the third quartile (Q3; top), the boundaries of the whiskers are evaluated by summing to the nearest quartile 1.5 times the interquartile range defined as Q3-Q1, and the dots represent the outliers from the whisker defined range.

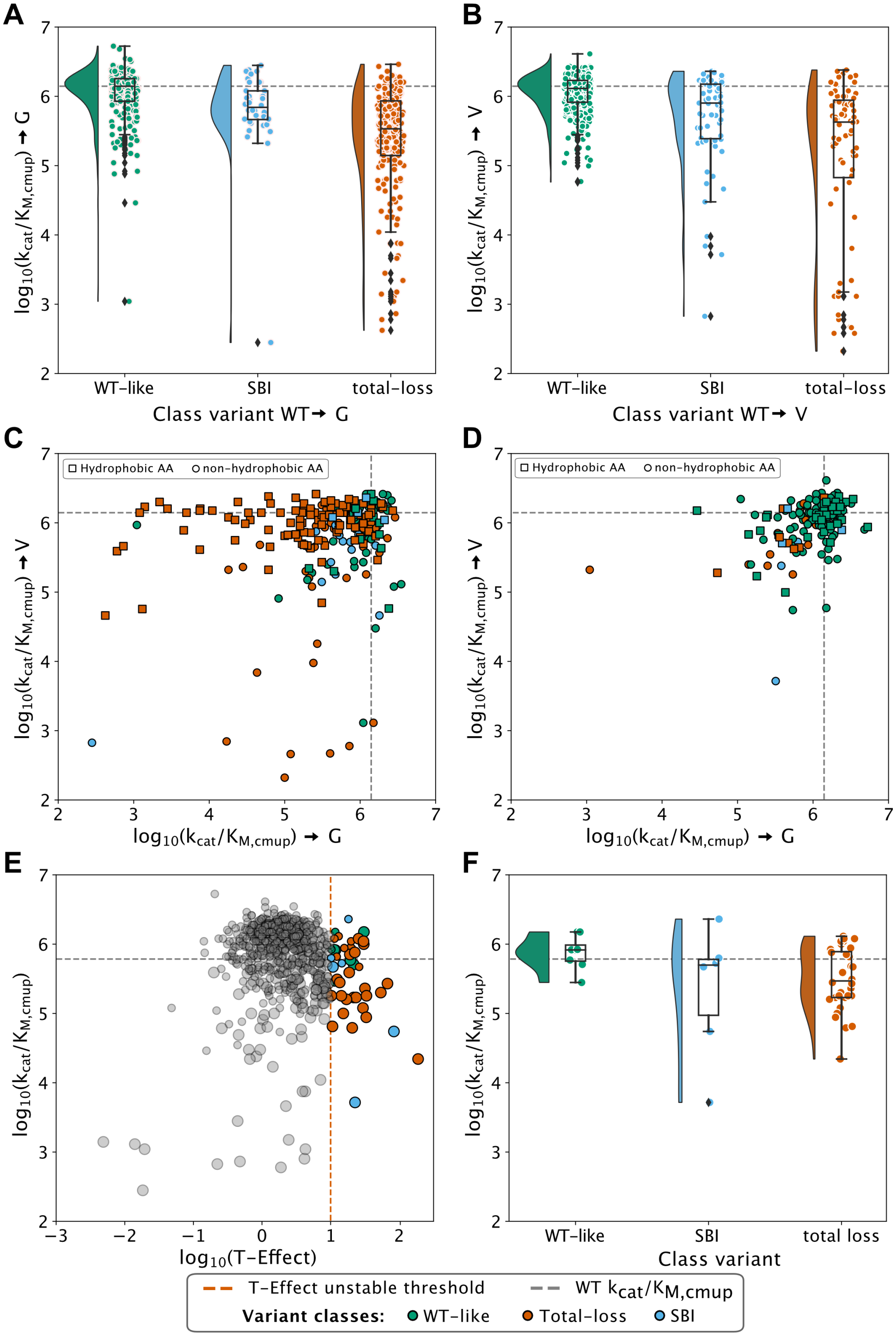

Supplementary Figure 5. Comparison of experiments and predictions for PafA (Uniprot: Q9KJX5). Raincloud plots for experimentally determined $k_{cat}/K_{M,cMUP}$ values when the wild-type amino acid was was substituted for (A) glycine or (B) valine. In both A and B, the values are shown separately for the different classes predicted by our model for the analysis of 488 mutations WT to glycine (A) and 470 substitutions WT to valine (B). The box plots shows the median and quartiles for each set of variants. The dashed horizontal lines show $k_{cat}/K_{M,cMUP}$ for WT PafA. (C, D) Scatter plot of $k_{cat}/K_{M,cMUP}$ values when substituting a residue with either glycine or valine. (C) Shows the comparison for buried residues (with an exposed surface area of less than 20%) and (D) shows exposed residues. In both C and D squared markers represent positions where the WT residue is hydrophobic and circles indicate non-hydrophobic WT residues. (E) Comparison of experimental $k_{cat}/K_{M,cMUP}$ values and temperature effects during expression. In particular, the T-effect value represents the change in measured catalytic efficiency when the protein was expressed at 23 °C or 37 °C (but with the enzymatic assay performed at 23 °C in both cases). Variants that show a substantial (greater than 10-fold) change nearly all belong to the total-loss category. Points with larger markers represent data for which the T-effect might be underestimated due to experimental limitations. (F) Raincloud plots for the 46 coloured variants highlighted in in panel E (large T-effect). In each boxplot, the central line represents the median values, the boundaries of the box represent the first quartile (Q1; bottom) and the third quartile (Q3; top), the boundaries of the whiskers are evaluated by summing to the nearest quartile 1.5 times the interquartile range defined as Q3-Q1, and the dots represent the outliers from the whisker defined range.

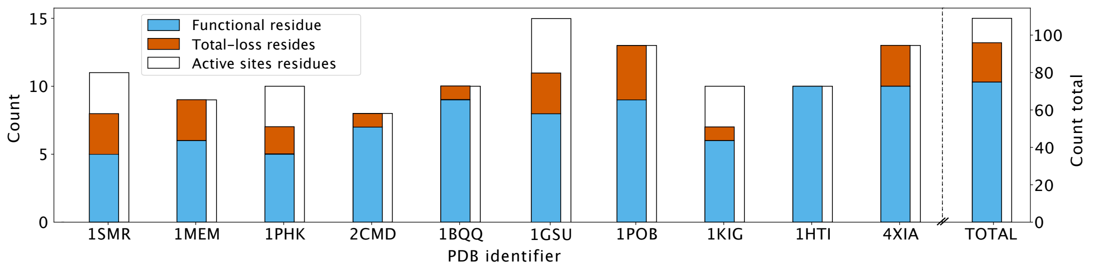

Supplementary Figure 6. Prediction of active site residues for the set of ten enzymes from [43]. The figure shows for each enzyme in the dataset the total number of reported active site positions (white), the subset of these predicted as being functional residues by our model (blue) and the positions predicted to be total loss (red). The rightmost bar shows the cumulative data.

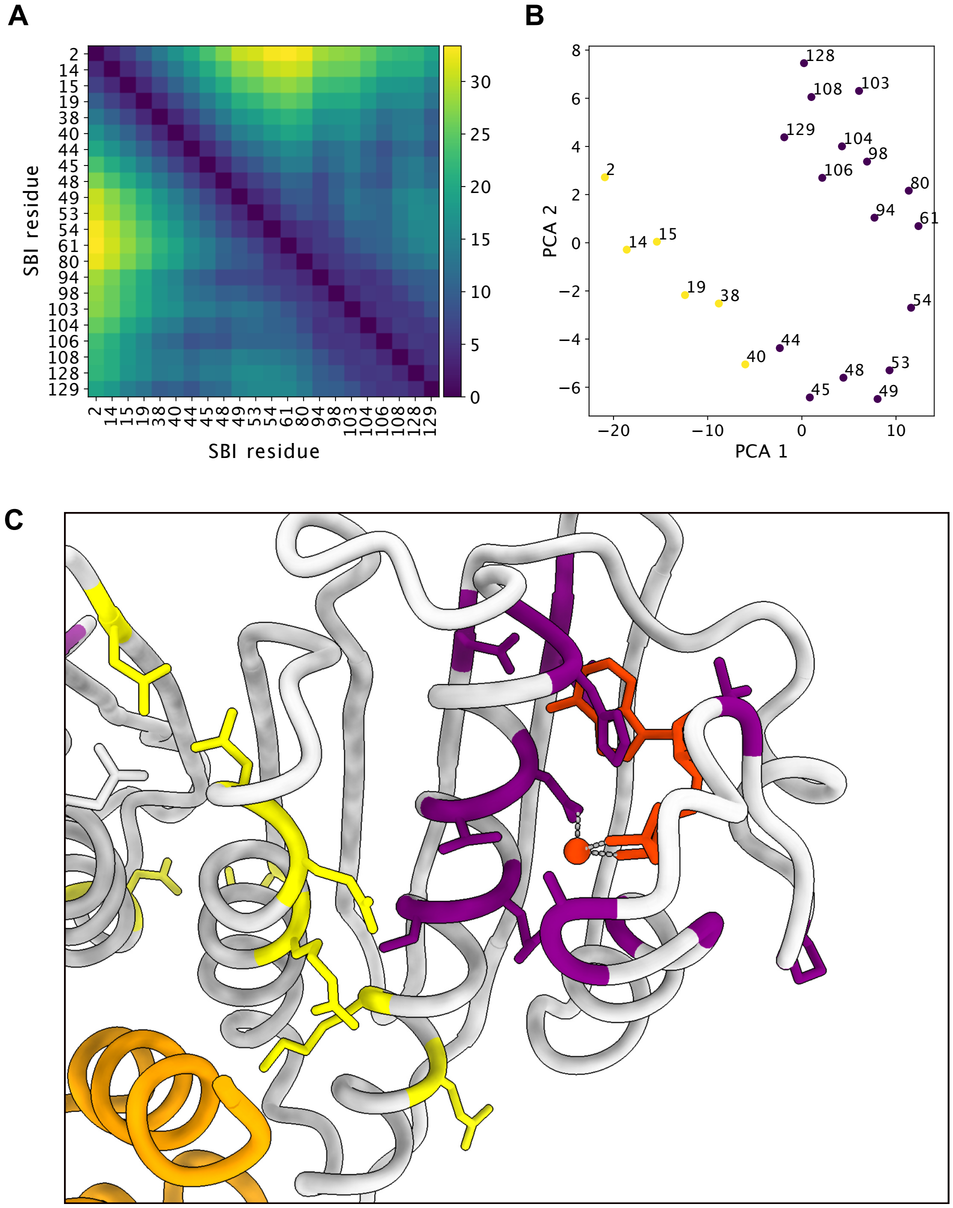

Supplementary Figure 7. Clusters of functional positions in Anti-Sigma factor. (A) C_α distance matrix for the positions classified as functional by our model. (B) A principal component analysis of the distance matrix suggests that the predicted functional sites can roughly be separated into two spatial clusters (coloured in yellow and purple along the first two principal components). (C) Functional sites coloured using the clustering.

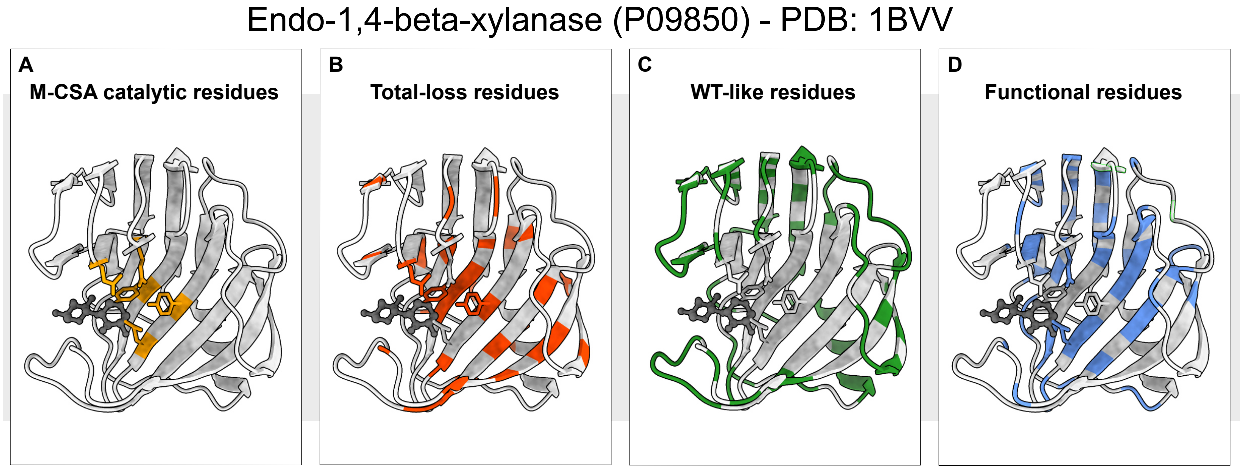

Supplementary Figure 8. Model predictions for Endo-1,4-beta-xylanase. (A) Catalytic residues in Endo-1,4-beta-xylanase as assigned by M-CSA. (B, C and D) show the residues which are classified by our model as (B) total loss, (C) wild-type like, and (D) functional residues. Our model predicts that substitutions at all five catalytic residues affect the protein function rendering it inactive. Three of the five positions are predicted to be important for function, but not for protein stability, whereas two positions are predicted to be important for both function and stability.

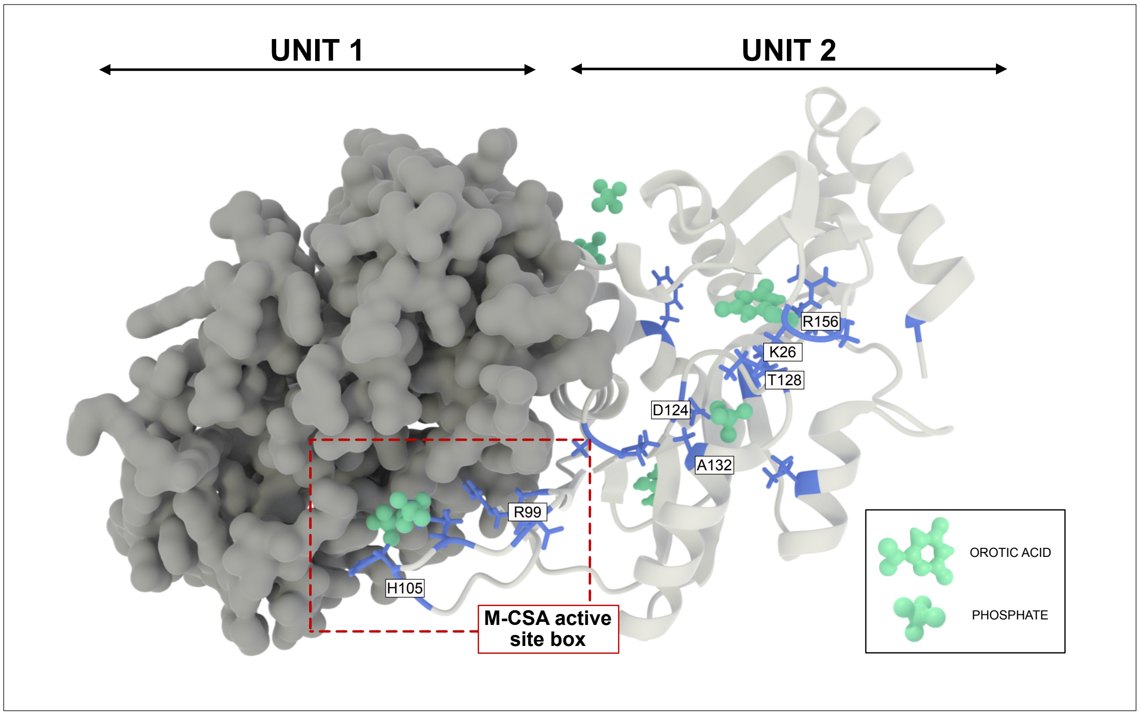

Supplementary Figure 9. Location of predicted functional sites in OPRTase. The figure shows the dimer structure of OPRTase, with the two sub-units coloured in grey and white, respectively. The residues classified as functional by our model are coloured in blue and labelled on the second sub-unit. The region surrounded by a dotted red box contains catalytic residues reported in the M-CSA database (again shown for the second sub-unit). Orotic acid and phosphate ions bound to the OPRTase complex are coloured in green.

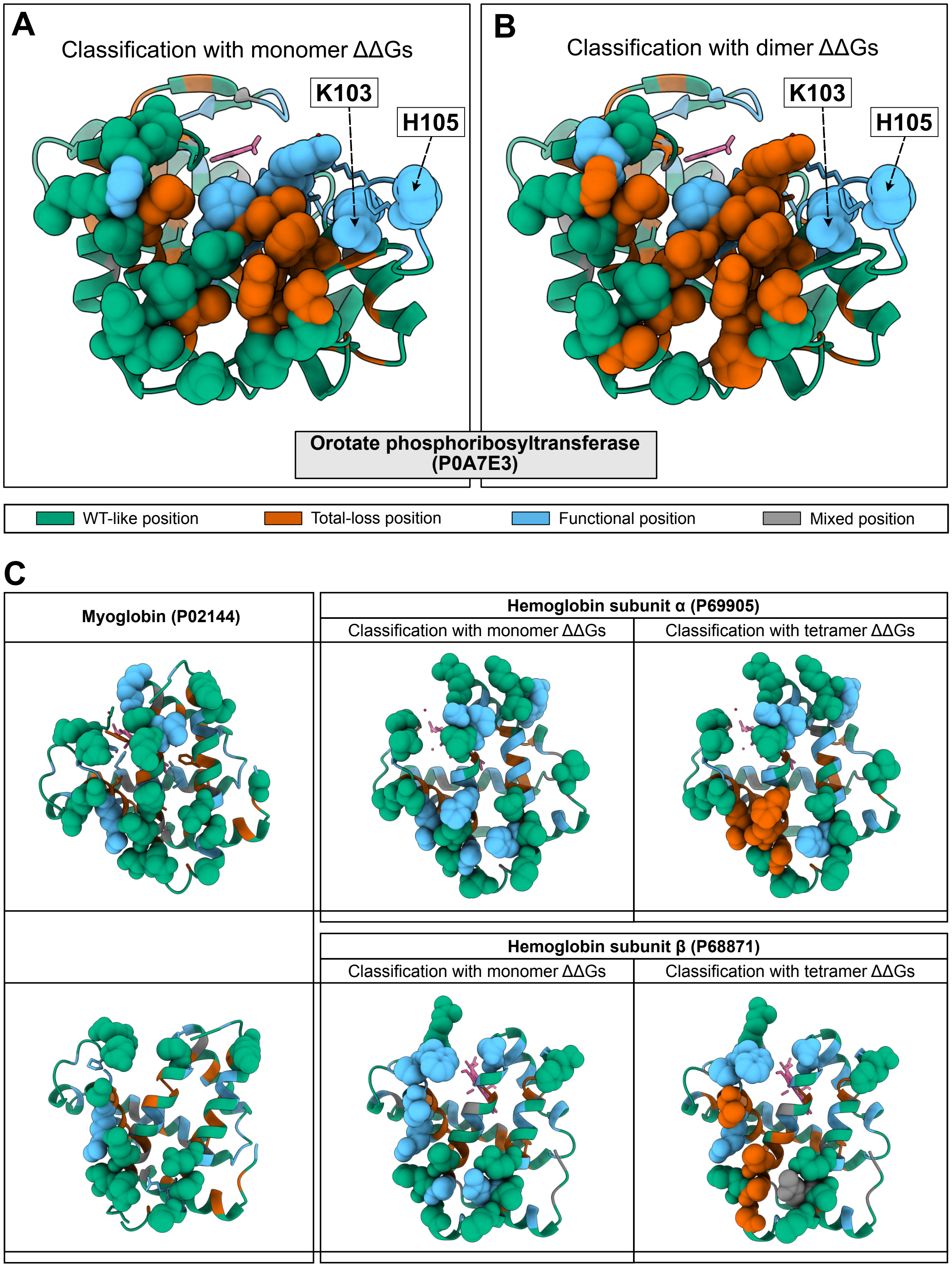

Supplementary Figure 10. Additional examples of the effect of input structure choice on residue classification in oligomeric proteins. Panels A and B show differences of classification for residues in orotate phosphoribosyltransferase when we use either (A) the monomer or (B) dimer structure as input to the Rosetta $\Delta\Delta G$ calculations. Residues at the interface are shown with van der Walls atomic representation and residues involved in forming the active site at the dimer interface are labelled. Panel C shows, like panel A, a comparison of predictions for human myoglobin and the α and β subunits of human hemoglobin. For human hemoglobin, the left column shows the residue classification using $\Delta\Delta G$ from the monomer, while the right column the classification made with $\Delta\Delta G$ keeping the entire tetrameric structure during the evaluation. Residues at the tetramer interface are shown with van der Walls atomic representation in all the hemoglobin panels; residues at the corresponding positions in myoglobin are highlighted to make comparisons easier.

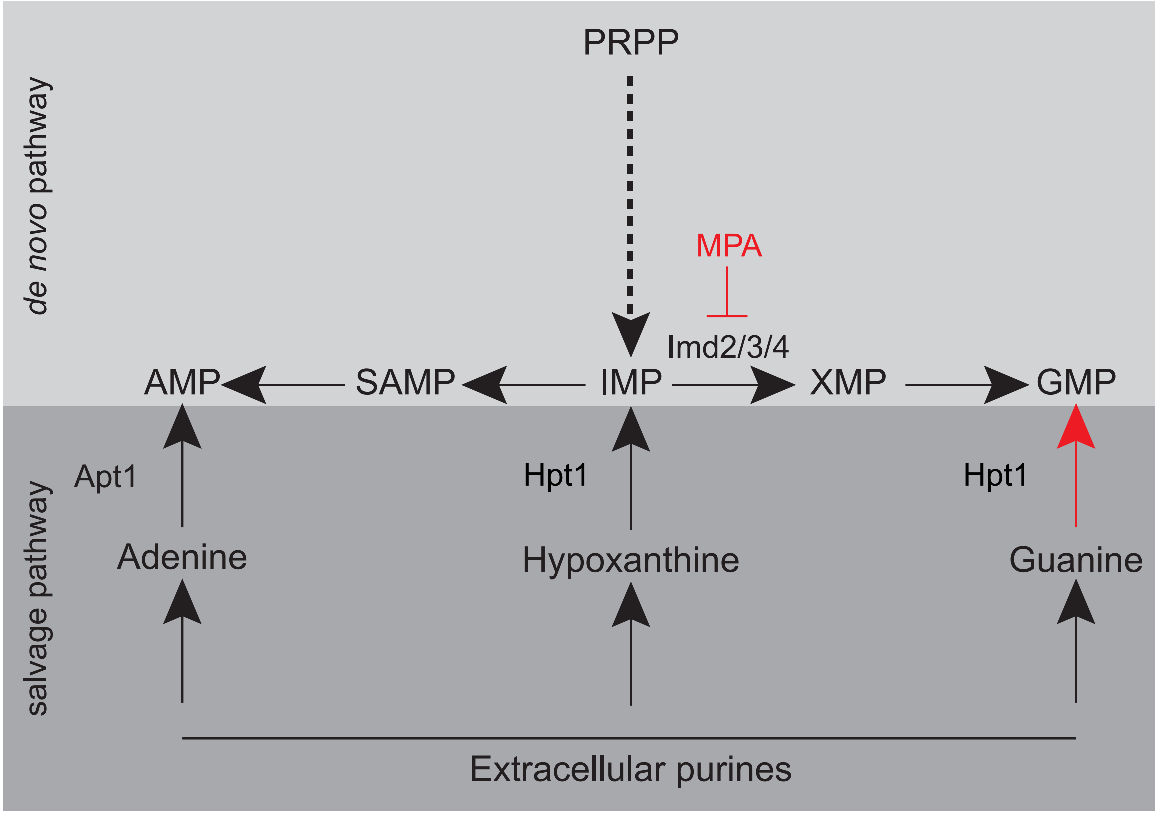

Supplementary Figure 11. *Hpt1* is essential in the presence of MPA. The figure shows relevant parts of yeast nucleotide metabolism including the salvage pathway where *Hpt1* is essential for generating GMP when XMP synthesis is blocked by MPA. A *hpt1*Δ yeast strain can therefore not grow in the presence of MPA, but can be rescued by human HPRT1 or functional HPRT1 variants.

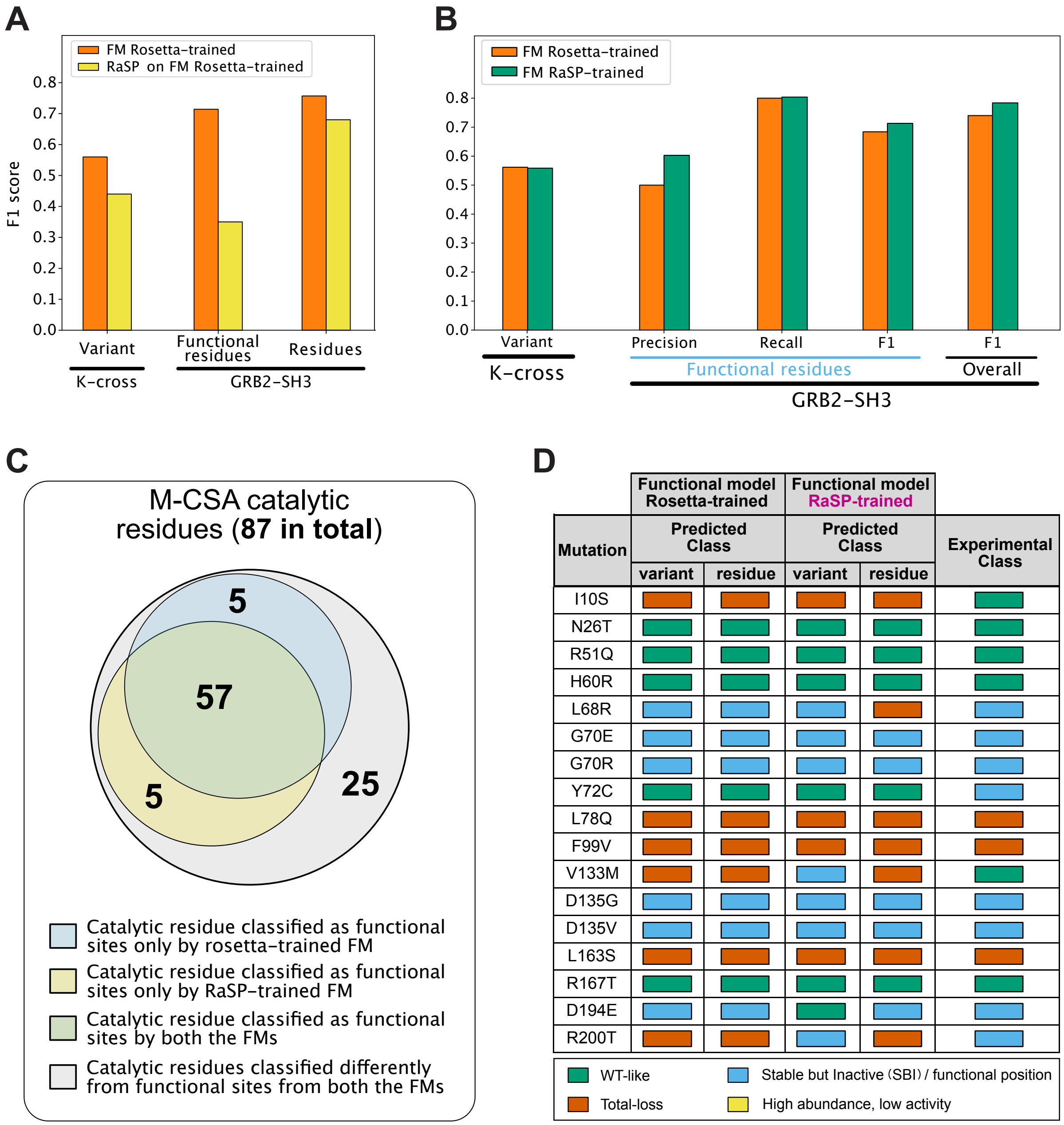

Supplementary Figure 12. Training a model for functional sites using thermodynamic stability changes predicted by RaSP. (A) Effects of using RaSP $\Delta\Delta G$ predictions as input on the functional residue model trained with Rosetta $\Delta\Delta G$ values. The leftmost set reports the F1 score on the test set during cross validation using as $\Delta\Delta G$ from Rosetta or RaSP. The two rightmost sets show the prediction performances on the GRB2 SH3 domain, for both the functional subset and over all the positions. (B) Comparing the performance of functional residue models trained using $\Delta\Delta G$ data from Rosetta (in orange) and RaSP (in green). The leftmost set reports the F1 score for the cross validation on the training data. The performance on the SH3 domain are shown in the remaining sets. (C) Venn diagram comparing the residues in the M-CSA set predicted from the two models. (D) Predictions of variant and residue classes by the two models are compared to the experimental results on the HPRT1 (rightmost column).

2. TABLES

Supplementary Table 1. Benchmarking models generated with reduced training sets. We trained vanilla models using 1%, 10% and 50% of the set of variants in NUDT15, PTEN and CYP2C9, and evaluated them on the held-out data in these three proteins and on the independent data for the SH3 domain of GRB2 (labelled SH3). All entries are F1 scores; the first entry is relate to NUDT15, PTEN and CYP2C9 and the last two relate to the GRB2 SH3 domain.

	Percentage variants in training dataset			
	1%	10%	50%	100%
Functional variants not used in training	0.24 + 0.05	0.34 +0.1	0.53+0.01	/
Functional variants in SH3	0.15+0.1	0.16 +0.08	0.25+0.03	0.32
All variants in SH3	0.40 + 0.12	0.61 + 0.04	0.62 + 0.01	0.63

Supplementary Table 2. Benchmarking models trained on two of the three proteins. We trained vanilla models using two of the three proteins (NUDT15, PTEN and CYP2C9), and evaluated them on the held-out protein and on the independent data for the SH3 domain of GRB2 (labelled SH3). All entries are F1 scores; the first two entries relate to NUDT15, PTEN and CYP2C9 and the last four relate to the GRB2 SH3 domain.

	Proteins in training dataset			
	NUDT15/CYP2C9	NUDT15/PTEN	CYP2C9/PTEN	ALL
Functional variants not used in training	0.13	0.33	0.27	/
Functional residues not used in training	0.58	0.51	0.57	/
Functional variants in SH3	0.19	0.30	0.25	0.32
All variants in SH3	0.62	0.62	0.60	0.63
Functional sites in SH3	0.36	0.35	0.40	0.60
All sites in SH3	0.72	0.71	0.69	0.75

Supplementary Table 3. Number of variants per class predicted by our model on the training set

Protein	WT-like	SBI	Total loss	Low abundance, high activity
NUDT15	1824 (68%)	350 (13%)	509 (19%)	1 (<1%)
PTEN	1689 (54%)	662 (21%)	755 (24%)	0 (0%)
CYP2C9	2396 (57%)	1111 (26%)	657 (17%)	0 (0%)

Supplementary Table 4. Enzymes analysed from the Mechanism and Catalytic Site Atlas database

Uniprot ID	PDB	Chain	# Analysed residues	# Catalytic residues	# Functional residues
Q9LAK3	1RO7	A	291	1	16
P82385	1DO6	A	124	2	11
P09850	1C5H	A	213	3	39
P56868	1B73	A	254	6	17
P0A7E3	1ORO	A	213	2	17
Q13569	3UFJ	A	410	1	26
P62593	1BTL	A	286	5	10
P00374	1DHF	A	187	1	9
P00469	1LCB	A	316	3	25
P00491	1RR6	A	289	3	16
Q06241	1R44	A	202	5	11
P04036	1ARZ	A	273	2	25
P0A6L0	1P1X	A	259	3	19
P19120	1KAZ	A	650	3	19
P77836	1BRW	A	433	5	35
Q16854	2OCP	A	277	2	17
Q53547	1AUO	A	219	3	9
Q55012	1PS1	A	337	6	8
Q8VQN0	1JC5	A	148	3	10

Supplementary Table 5. Proteins analysed from the Protein-Protein Interaction Affinity Database 2.0

Uniprot ID	PDB	Chain	# Analysed residues	# SBI residues
P0AE67	1FFW	A	129	6
P01588	1EER	A	193	17
P61972	1A2K	A	127	19
P0A0L5	3BZD	B	237	7
O77044	1KSD	A	433	12

Supplementary Table 6. Information on proteins used in the manuscript which are not included in the previous tables

Protein	Uniprot ID	PDB	Chain
Nucleotide triphosphate diphosphatase (NUDT15)	Q9NV35	5BON	A
PTEN	P60484	1D5R	A
Cytochrome P450 2C9 (CYP2C9)	P11712	1OG2	A
Alkaline phosphatase PafA	Q9KJX5	5TJ3	A
Growth factor receptor-bound protein 2 (GRB2)	P62993	AlphaFold	A
Disks large homolog 4 (DLH4)	P78352	6QJJ	A
Hypoxanthine-guanine phosphoribosyltransferase (HPRT1)	P00492	1Z7G	A
Anti-signa F factor	O32727	1L0O	A
