# Supplementary material for: Discovering functionally important sites in proteins
Source: Nat Commun. 2023 Jul 13;14:4175. doi: 10.1038/s41467-023-39909-0 (PMC10345196; doi:10.1038/s41467-023-39909-0)
Supplement: Supplementary file 3 — Reporting Summary [file 41467_2023_39909_MOESM3_ESM.pdf]

nature portfolio

Corresponding author(s): Kresten Lindorff-Larsen & Rasmus Hartmann-Petersen

Last updated by author(s): Jun 15, 2023

Reporting Summary

Nature Portfolio wishes to improve the reproducibility of the work that we publish. This form provides structure for consistency and transparency in reporting. For further information on Nature Portfolio policies, see our Editorial Policies and the Editorial Policy Checklist.

Statistics

For all statistical analyses, confirm that the following items are present in the figure legend, table legend, main text, or Methods section.

n/a	Confirmed	
☐	☒	The exact sample size (*n*) for each experimental group/condition, given as a discrete number and unit of measurement
☒	☐	A statement on whether measurements were taken from distinct samples or whether the same sample was measured repeatedly
☒	☐	The statistical test(s) used AND whether they are one- or two-sided<br>*Only common tests should be described solely by name; describe more complex techniques in the Methods section.*
☒	☐	A description of all covariates tested
☒	☐	A description of any assumptions or corrections, such as tests of normality and adjustment for multiple comparisons
☐	☒	A full description of the statistical parameters including central tendency (e.g. means) or other basic estimates (e.g. regression coefficient) AND variation (e.g. standard deviation) or associated estimates of uncertainty (e.g. confidence intervals)
☒	☐	For null hypothesis testing, the test statistic (e.g. *F*, *t*, *r*) with confidence intervals, effect sizes, degrees of freedom and *P* value noted<br>*Give P values as exact values whenever suitable.*
☒	☐	For Bayesian analysis, information on the choice of priors and Markov chain Monte Carlo settings
☒	☐	For hierarchical and complex designs, identification of the appropriate level for tests and full reporting of outcomes
☐	☒	Estimates of effect sizes (e.g. Cohen's *d*, Pearson's *r*), indicating how they were calculated

Our web collection on statistics for biologists contains articles on many of the points above.

Software and code

Policy information about availability of computer code

Data collection	Code, data and analysis scripts are available at https://github.com/KULL-Centre/_2022_functional-sites-cagiada
Data analysis	Codes and script used are available at https://github.com/KULL-Centre/_2022_functional-sites-cagiada.<br>Additional code that we used include:<br>MDTraj v.1.9.3<br>Rosetta (GitHub SHA1 99d33ec59ce9fcecc5e4f3800c778a54afdf8504)<br>RaSP v.1.0<br>GEMME v1.0<br>Catboost v.0.26.1<br>scikit-learn v.1.0.2<br>numpy v.1.21.5<br>scipy v.1.7.3<br>matplotlib v.3.5.3<br>ptitprince v.0.2.6

For manuscripts utilizing custom algorithms or software that are central to the research but not yet described in published literature, software must be made available to editors and reviewers. We strongly encourage code deposition in a community repository (e.g. GitHub). See the Nature Portfolio guidelines for submitting code & software for further information.

Data

Policy information about availability of data

All manuscripts must include a data availability statement. This statement should provide the following information, where applicable:

- Accession codes, unique identifiers, or web links for publicly available datasets
- A description of any restrictions on data availability
- For clinical datasets or third party data, please ensure that the statement adheres to our policy

The input data for the model, the functional prediction used in this study as well as the model binary are available on the Github of the project https://github.com/KULL-Centre/_2022_functional-sites-cagiada). Accession codes for the proteins used are available in Supplementary Table 4,5,6.

Research involving human participants, their data, or biological material

Policy information about studies with human participants or human data. See also policy information about sex, gender (identity/presentation), and sexual orientation and race, ethnicity and racism.

Reporting on sex and gender	NA
Reporting on race, ethnicity, or other socially relevant groupings	NA
Population characteristics	NA
Recruitment	NA
Ethics oversight	NA

Note that full information on the approval of the study protocol must also be provided in the manuscript.

Field-specific reporting

Please select the one below that is the best fit for your research. If you are not sure, read the appropriate sections before making your selection.

☒ Life sciences ☐ Behavioural & social sciences ☐ Ecological, evolutionary & environmental sciences

For a reference copy of the document with all sections, see nature.com/documents/nr-reporting-summary-flat.pdf

Life sciences study design

All studies must disclose on these points even when the disclosure is negative.

Sample size	No sample size calculation was performed as this was not relevant to the study
Data exclusions	No data were excluded
Replication	The yeast growth and western blots were repeated three times. All other experiments were computational.
Randomization	Randomization was used to train the ML model as described in the paper and associated code
Blinding	The ML model was tested using independent data as described in the paper

Reporting for specific materials, systems and methods

We require information from authors about some types of materials, experimental systems and methods used in many studies. Here, indicate whether each material, system or method listed is relevant to your study. If you are not sure if a list item applies to your research, read the appropriate section before selecting a response.

Materials & experimental systems

n/a	Involved in the study
☐	☒ Antibodies
☒	☐ Eukaryotic cell lines
☒	☐ Palaeontology and archaeology
☒	☐ Animals and other organisms
☒	☐ Clinical data
☒	☐ Dual use research of concern
☒	☐ Plants

Methods

n/a	Involved in the study
☒	☐ ChIP-seq
☒	☐ Flow cytometry
☒	☐ MRI-based neuroimaging

Antibodies

Antibodies used

The antibodies were mouse IgG1 anti-RGSHis (Qiagen, Cat. No. 34650) diluted 1:2000 and peroxidase-conjugated polyclonal rabbit anti-mouse antibody (Dako, Cat. No. P0260) diluted 1:5000.

Validation

The results shown in Figure 6 include both positive and negative controls. No reactivity was observed in lysates from a vector transformed control strain.
