# Supplementary material for: Discovering functionally important sites in proteins
Source: Nat Commun. 2023 Jul 13;14:4175. doi: 10.1038/s41467-023-39909-0 (PMC10345196; doi:10.1038/s41467-023-39909-0)

Upper panels

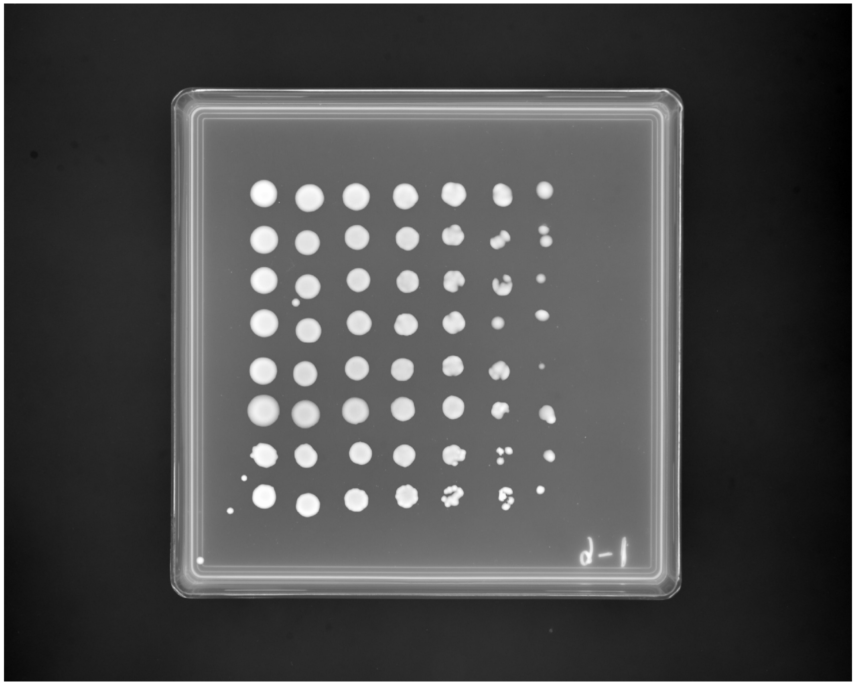

control

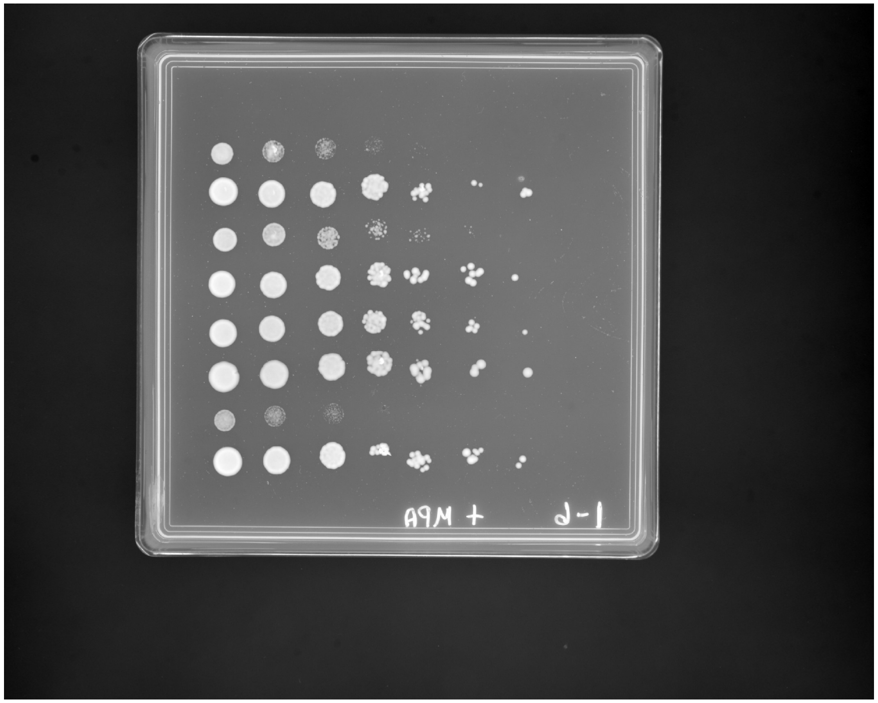

+MPA

Central panels

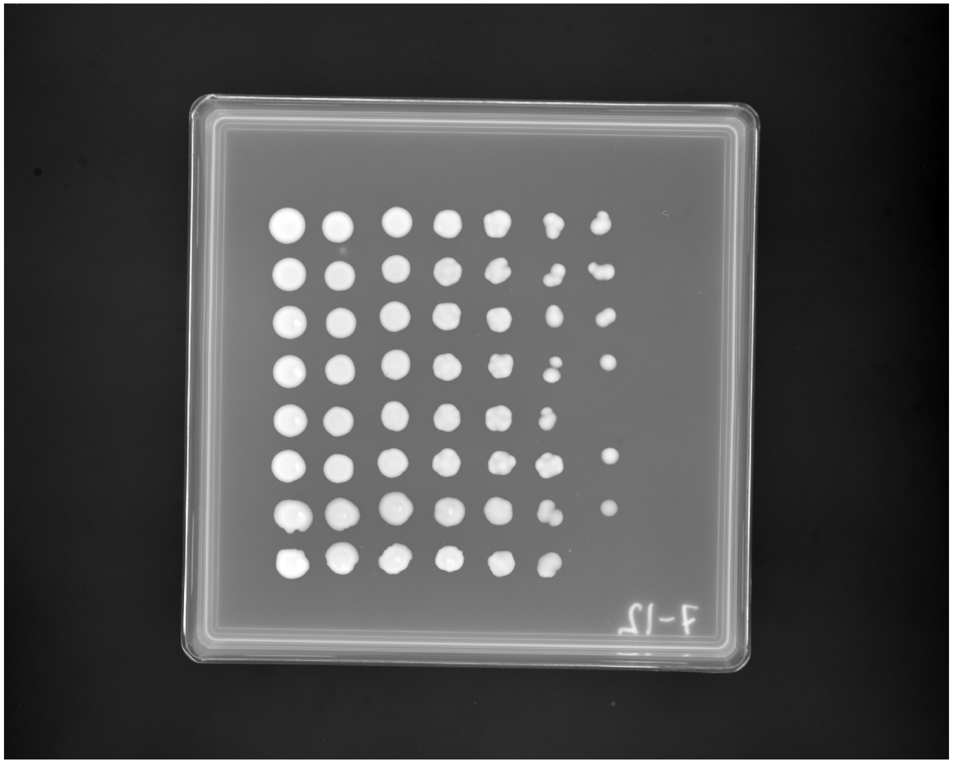

control

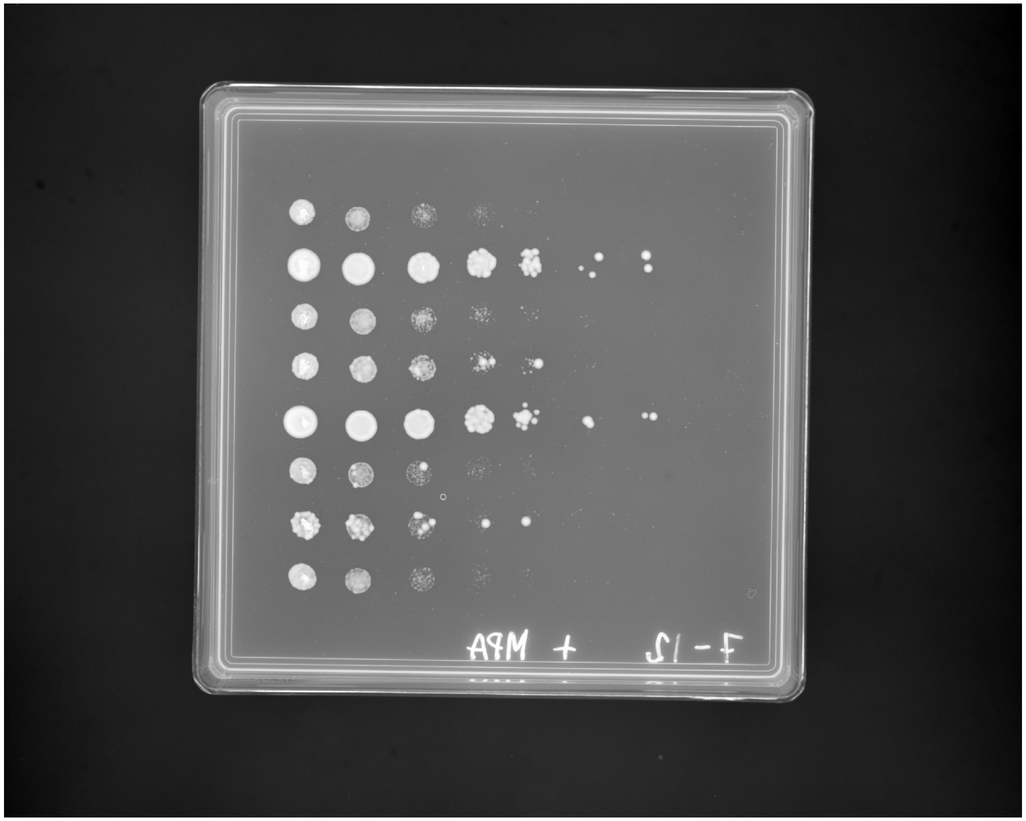

+MPA

Lower panels

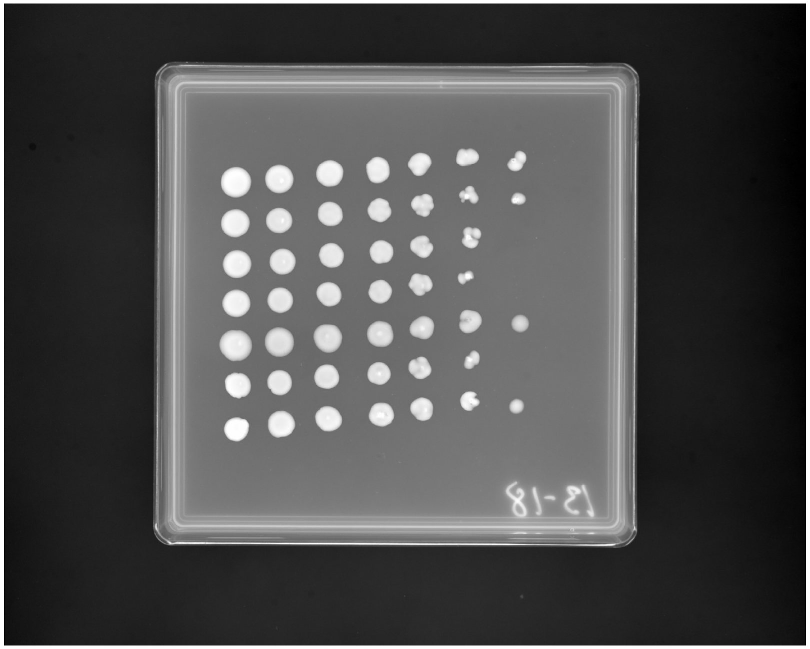

control

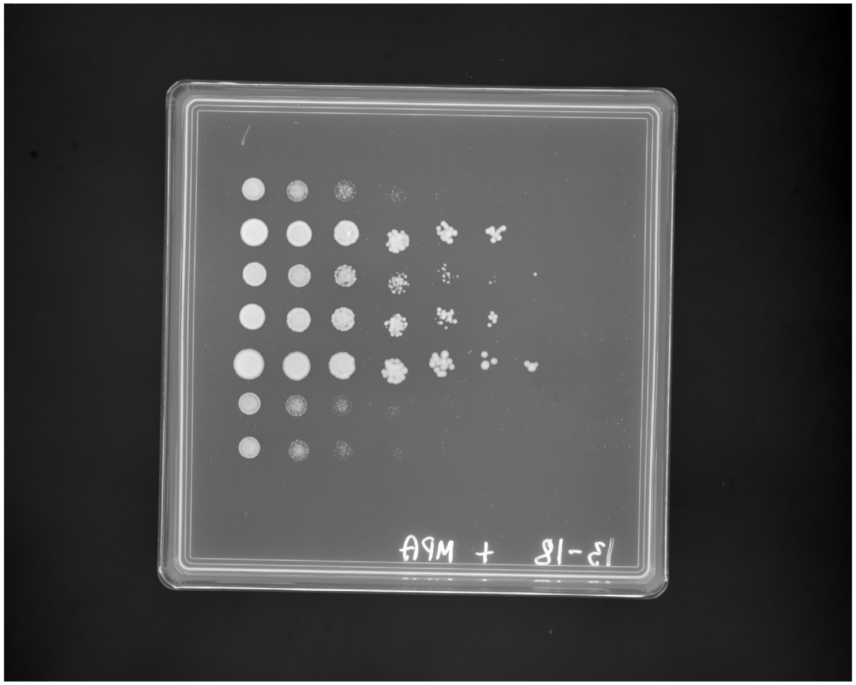

+MPA

Left panels

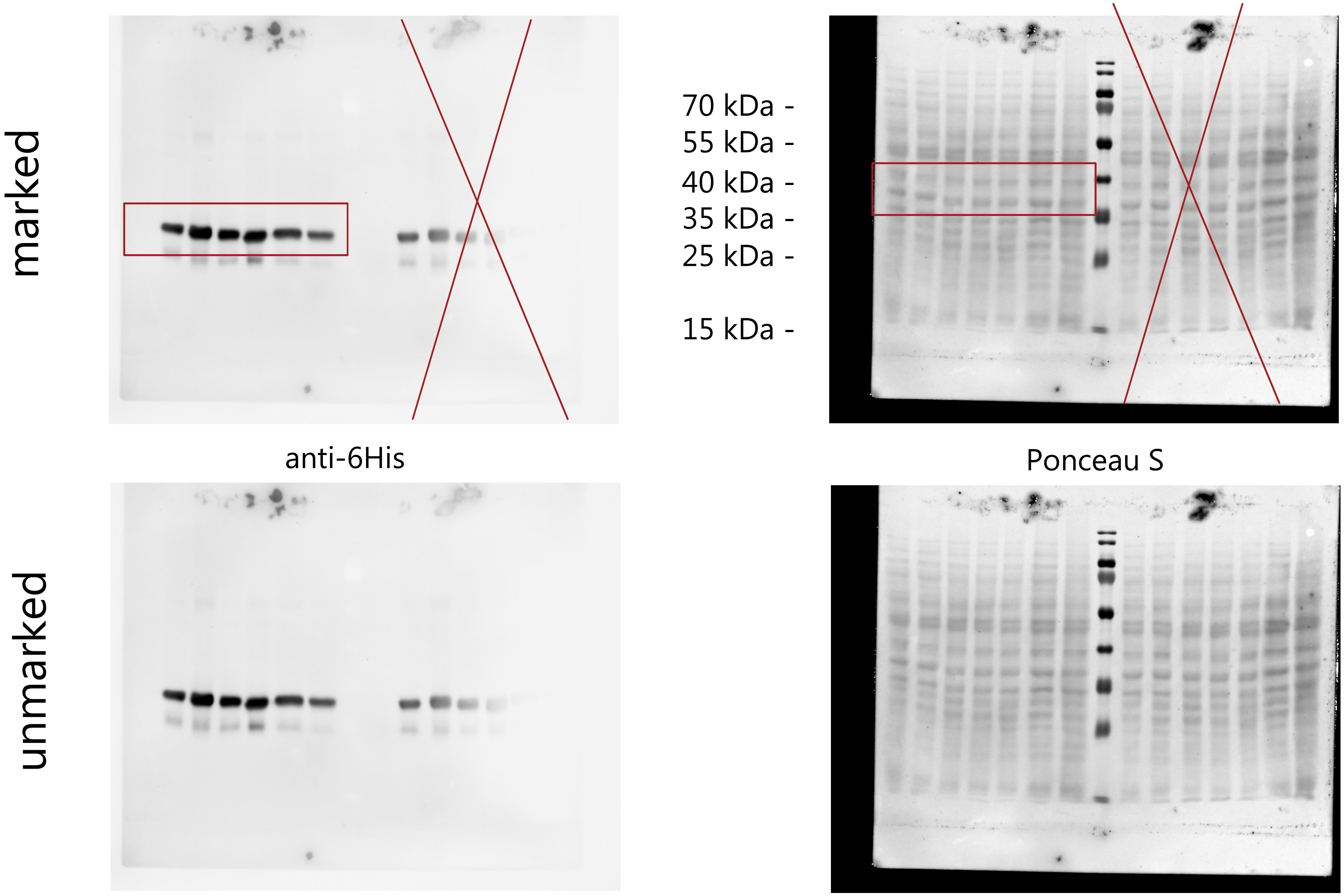
marked
unmarked
anti-6His
Ponceau S
70 kDa -
55 kDa -
40 kDa -
35 kDa -
25 kDa -
15 kDa -

Central panels

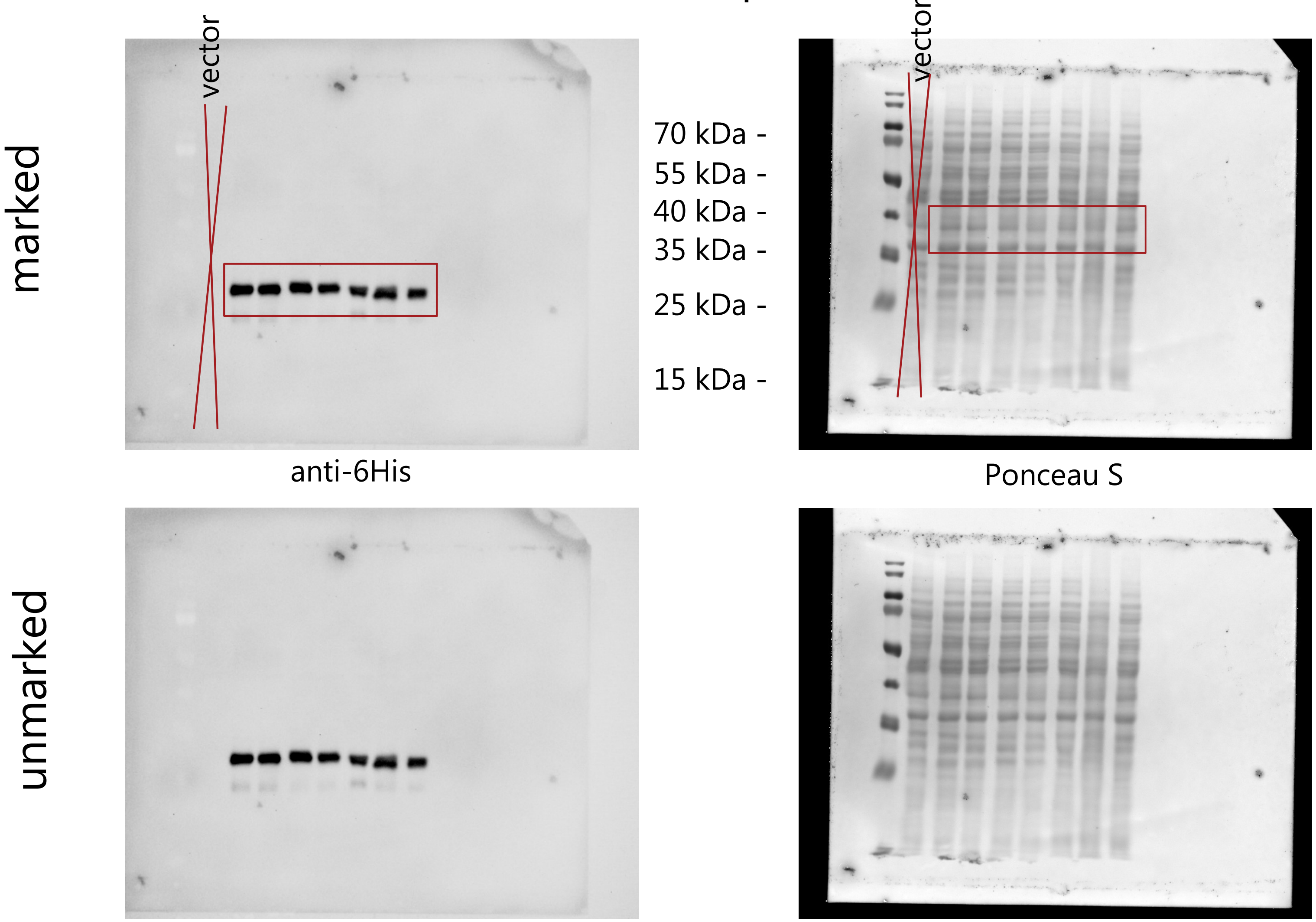

Right panels

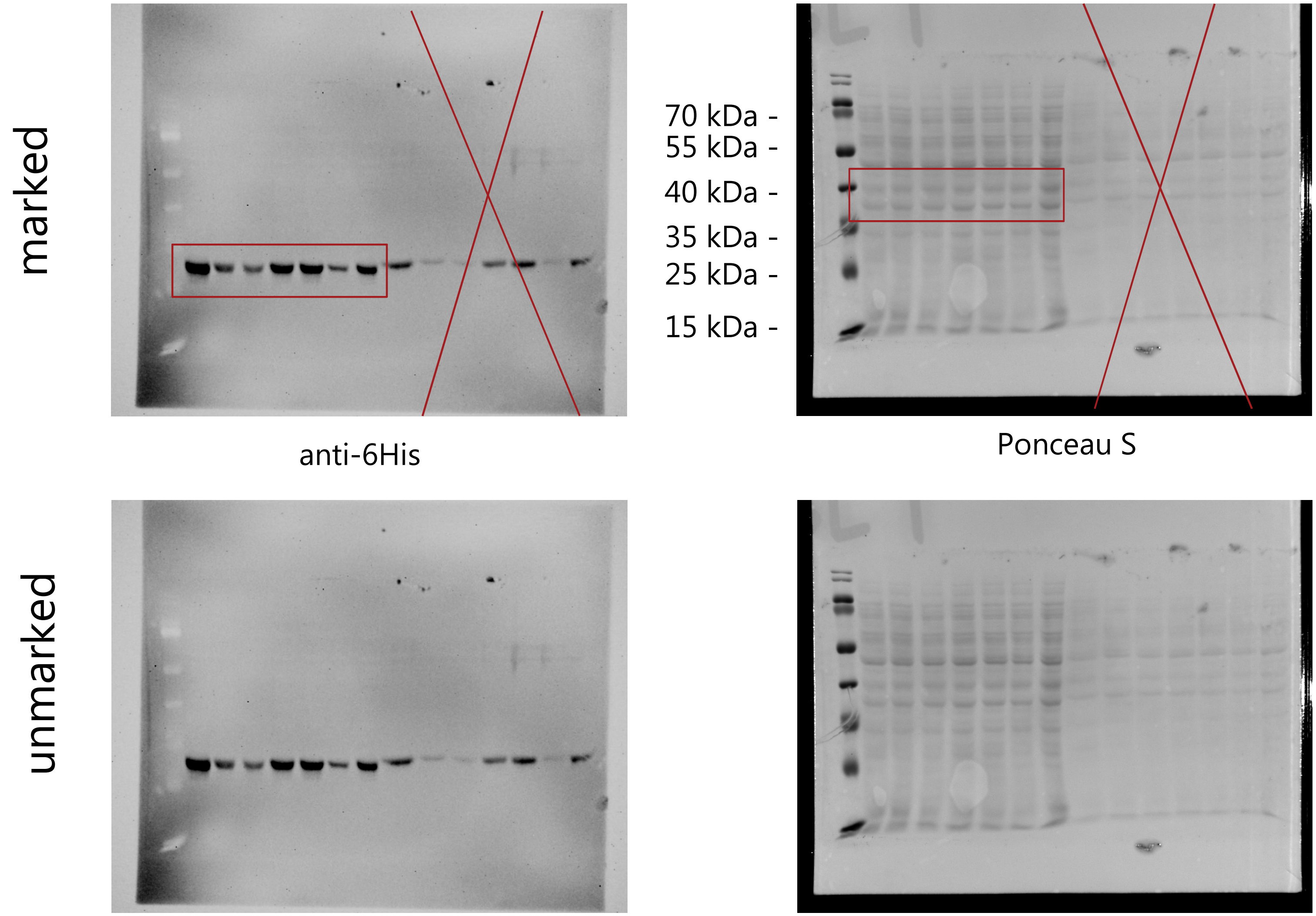

Supplement: Supplementary file 4 — Source Data [file 41467_2023_39909_MOESM4_ESM.zip › SourceData_NCOMMS_23_22106_Lindorff_Larsen/Figure_6/RawSpots_and_Westerns.pdf]
