# Supplementary figures and images for: Discovering functionally important sites in proteins
Source: Nat Commun. 2023 Jul 13;14:4175. doi: 10.1038/s41467-023-39909-0 (PMC10345196; doi:10.1038/s41467-023-39909-0)

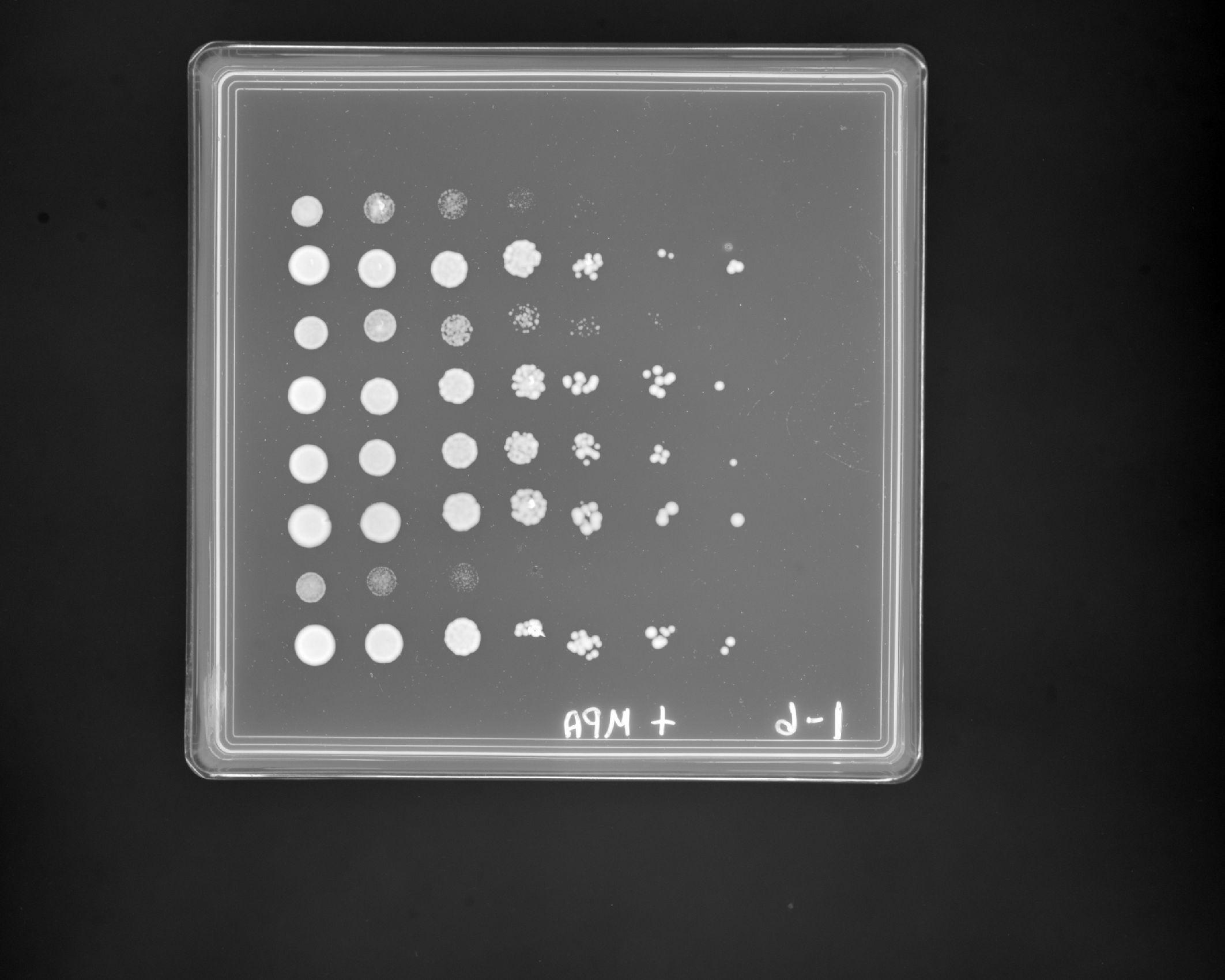

Supplement: Supplementary file 4 — Source Data [file 41467_2023_39909_MOESM4_ESM.zip › SourceData_NCOMMS_23_22106_Lindorff_Larsen/Figure_6/image14.tif]

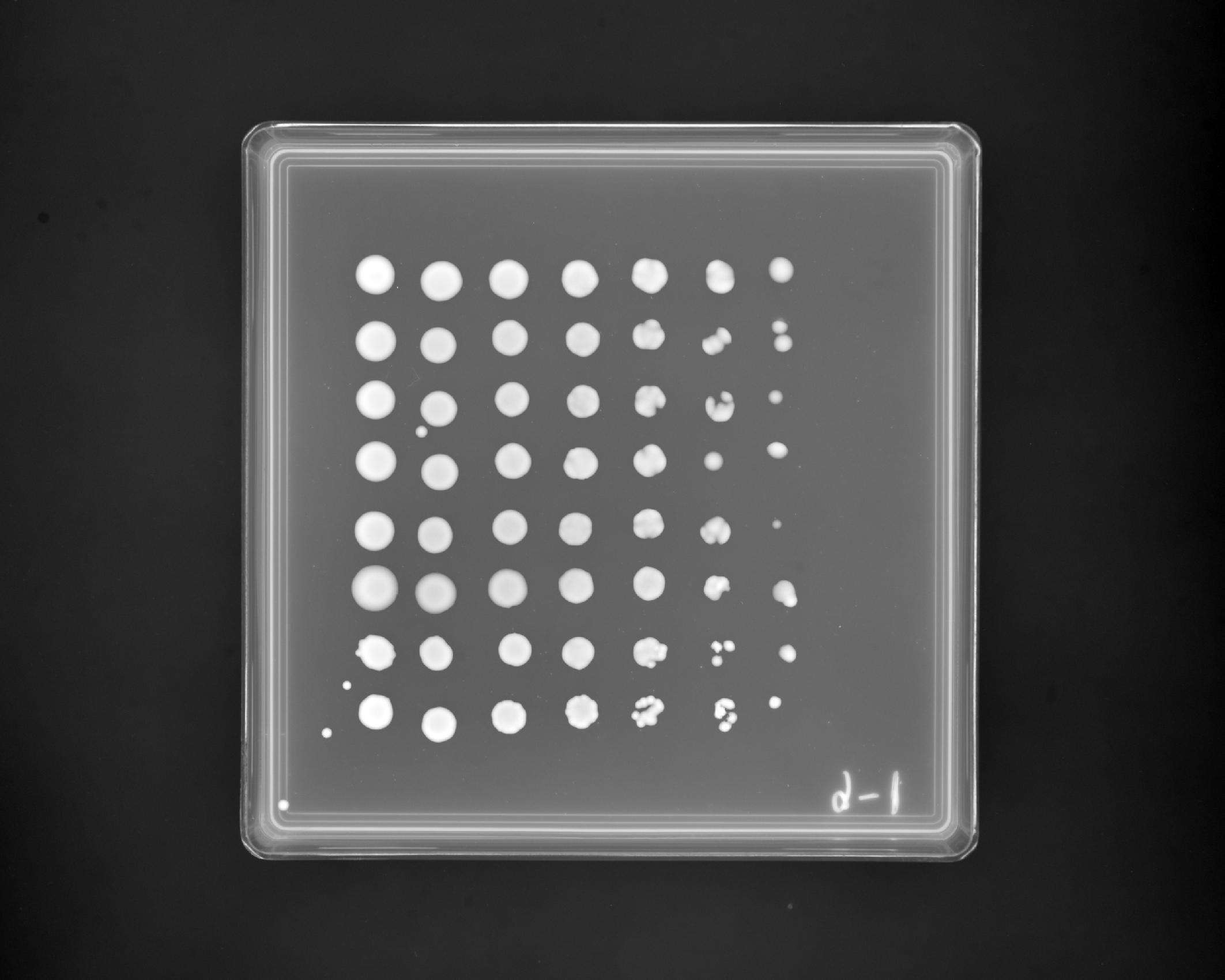

Supplement: Supplementary file 4 — Source Data [file 41467_2023_39909_MOESM4_ESM.zip › SourceData_NCOMMS_23_22106_Lindorff_Larsen/Figure_6/image15.tif]

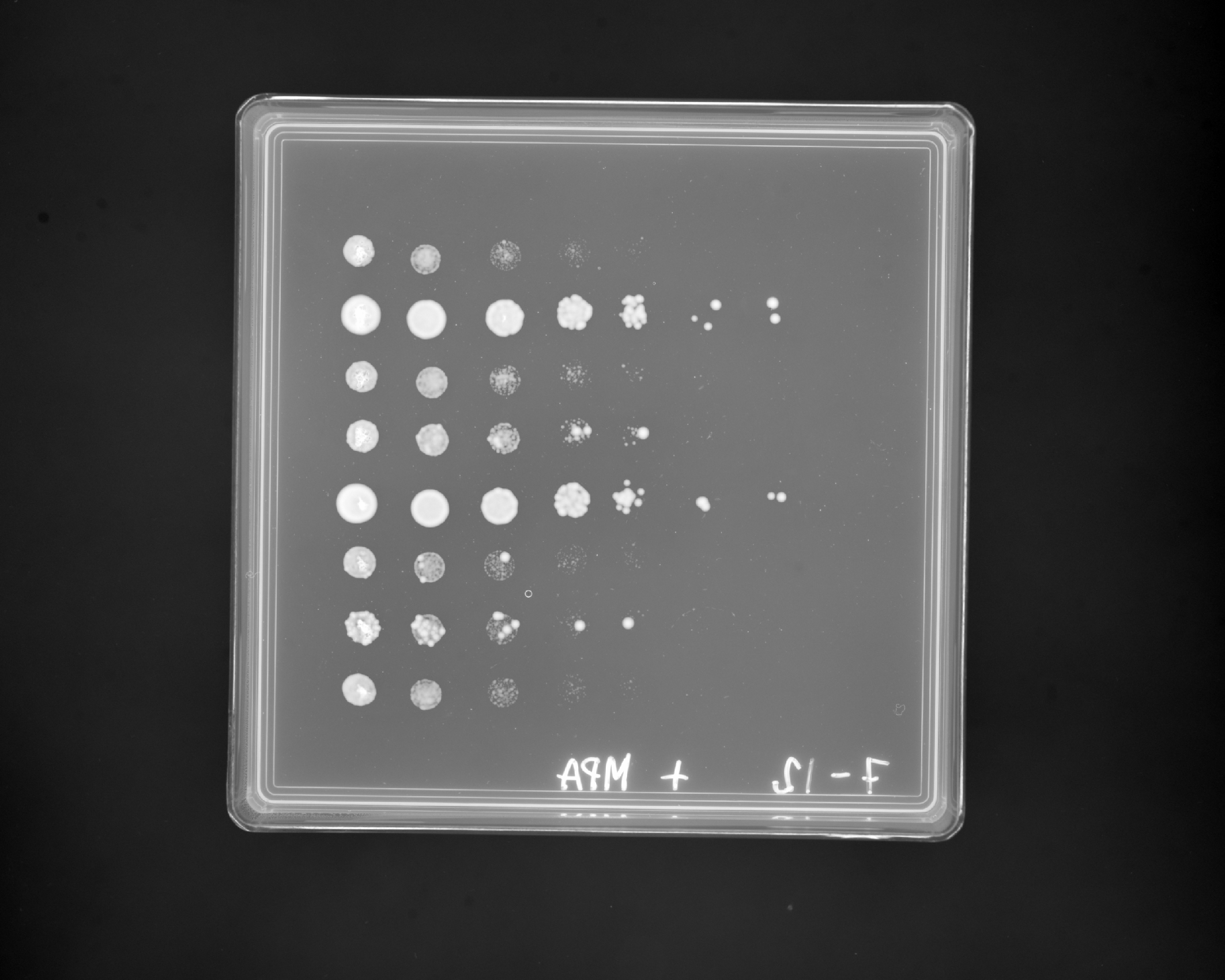

Supplement: Supplementary file 4 — Source Data [file 41467_2023_39909_MOESM4_ESM.zip › SourceData_NCOMMS_23_22106_Lindorff_Larsen/Figure_6/image16.tif]

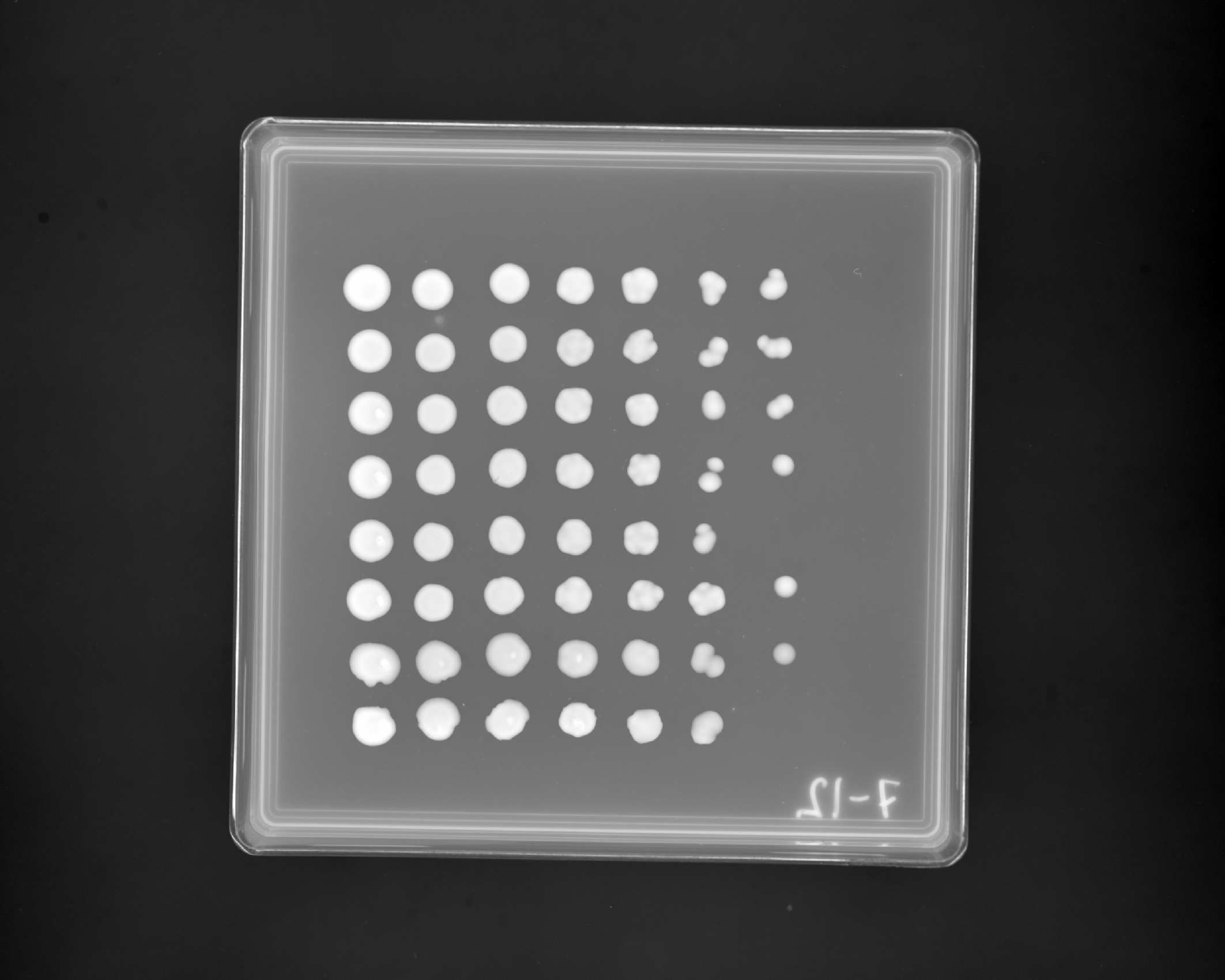

Supplement: Supplementary file 4 — Source Data [file 41467_2023_39909_MOESM4_ESM.zip › SourceData_NCOMMS_23_22106_Lindorff_Larsen/Figure_6/image17.tif]

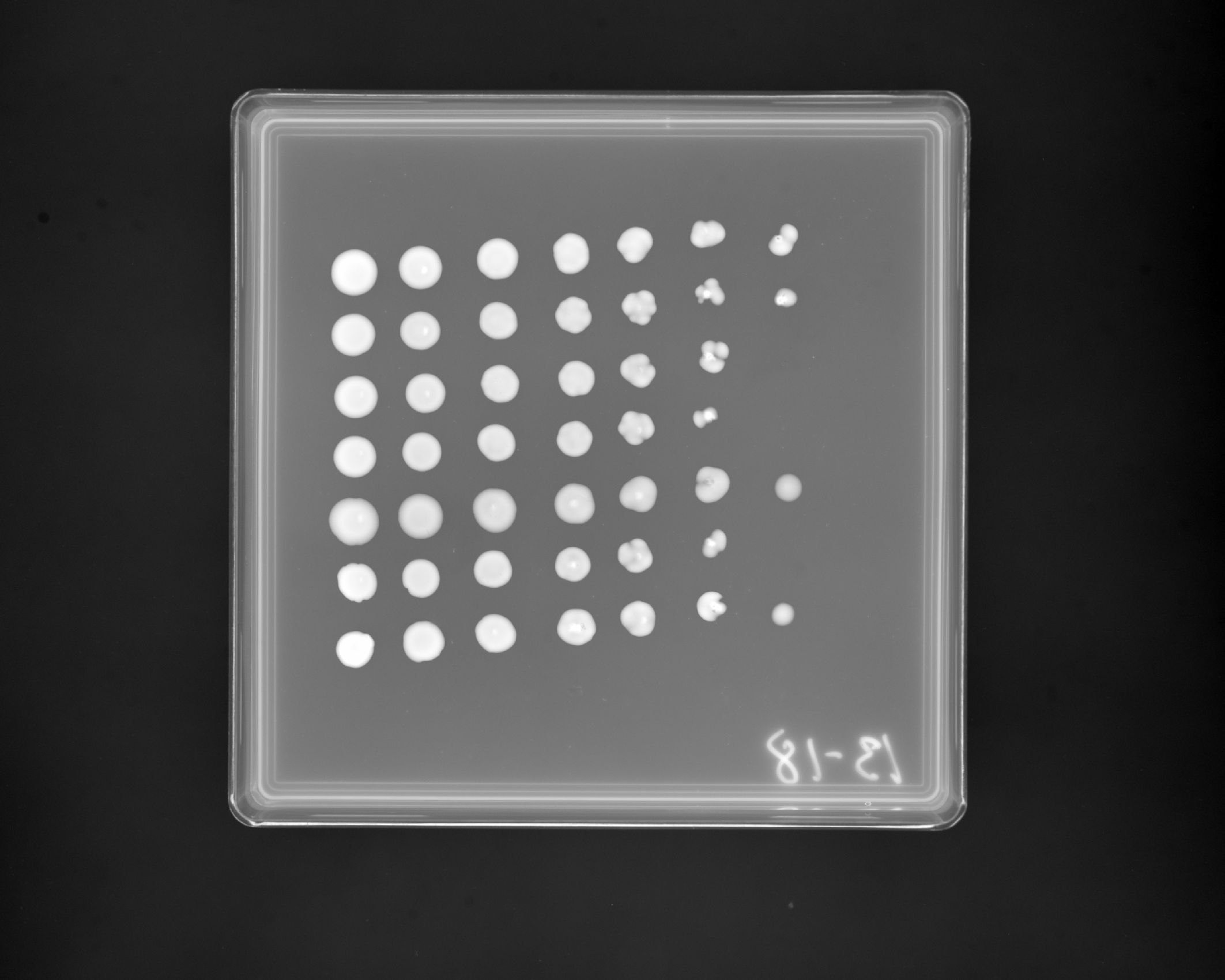

Supplement: Supplementary file 4 — Source Data [file 41467_2023_39909_MOESM4_ESM.zip › SourceData_NCOMMS_23_22106_Lindorff_Larsen/Figure_6/image18.tif]

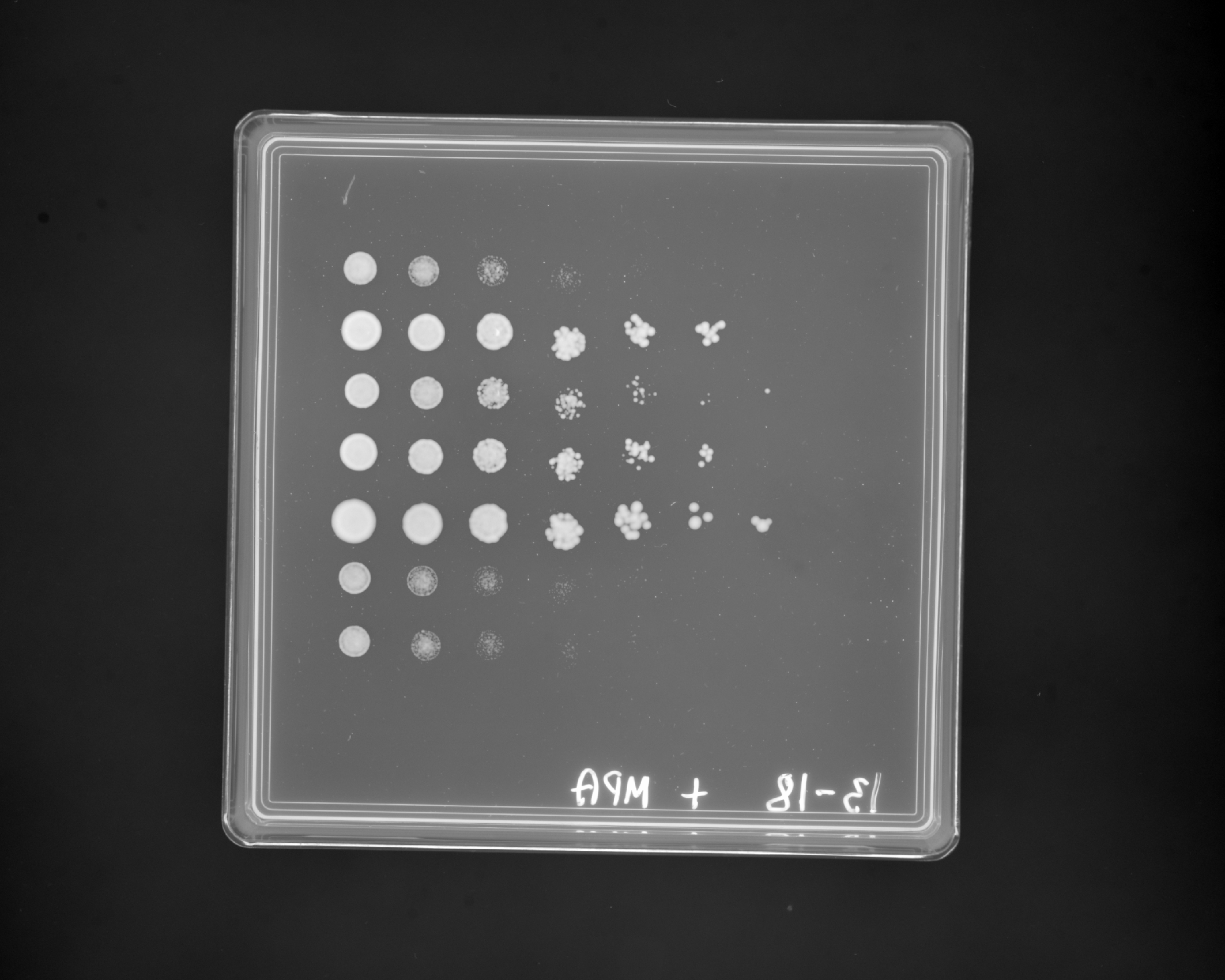

Supplement: Supplementary file 4 — Source Data [file 41467_2023_39909_MOESM4_ESM.zip › SourceData_NCOMMS_23_22106_Lindorff_Larsen/Figure_6/image19.tif]

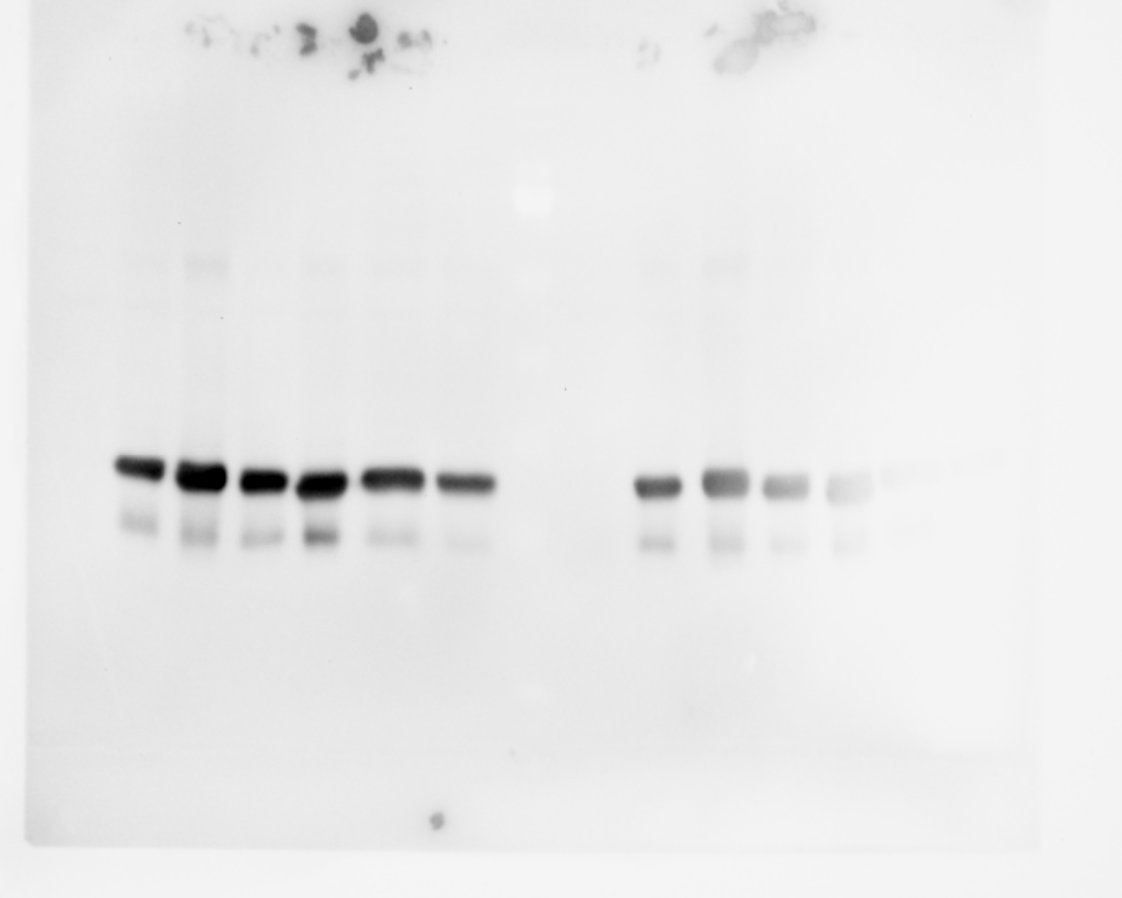

Supplement: Supplementary file 4 — Source Data [file 41467_2023_39909_MOESM4_ESM.zip › SourceData_NCOMMS_23_22106_Lindorff_Larsen/Figure_6/image20.tif]

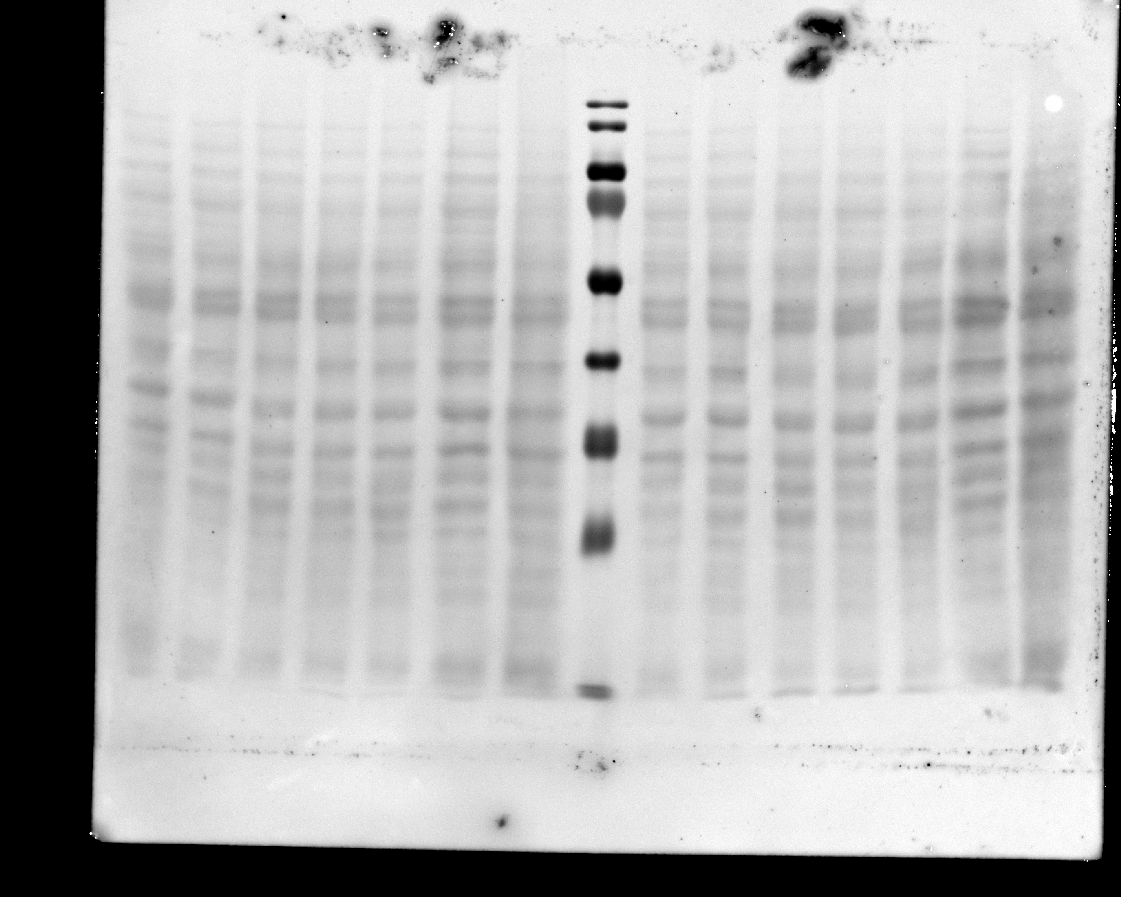

Supplement: Supplementary file 4 — Source Data [file 41467_2023_39909_MOESM4_ESM.zip › SourceData_NCOMMS_23_22106_Lindorff_Larsen/Figure_6/image21.tif]

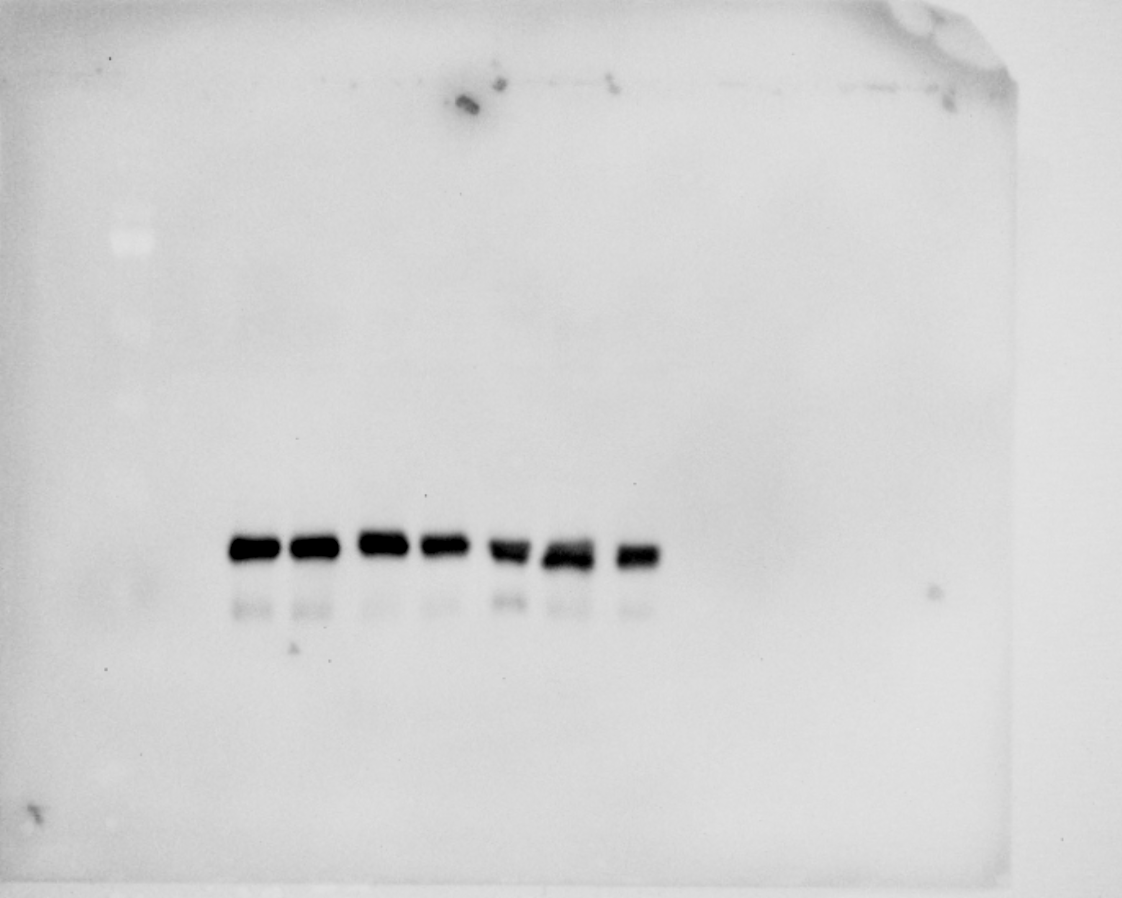

Supplement: Supplementary file 4 — Source Data [file 41467_2023_39909_MOESM4_ESM.zip › SourceData_NCOMMS_23_22106_Lindorff_Larsen/Figure_6/image22.tif]

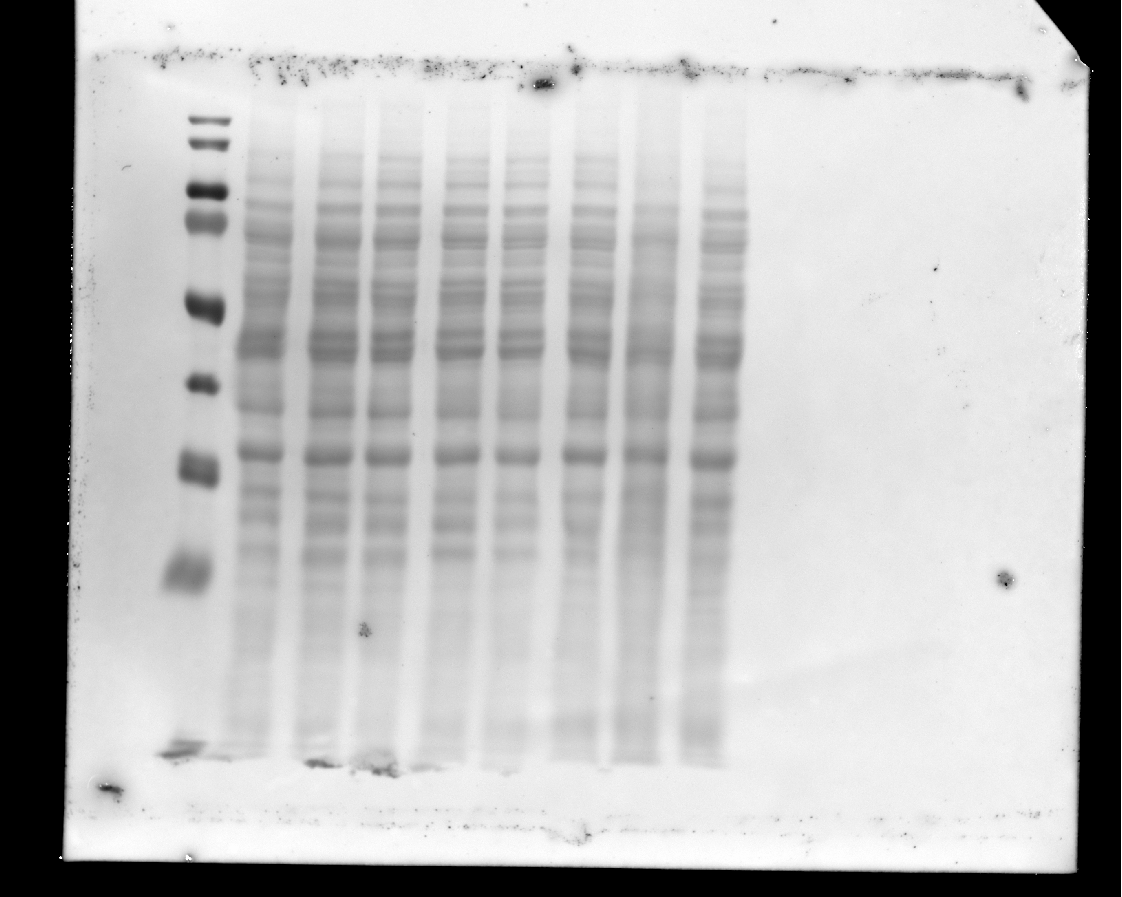

Supplement: Supplementary file 4 — Source Data [file 41467_2023_39909_MOESM4_ESM.zip › SourceData_NCOMMS_23_22106_Lindorff_Larsen/Figure_6/image23.tif]

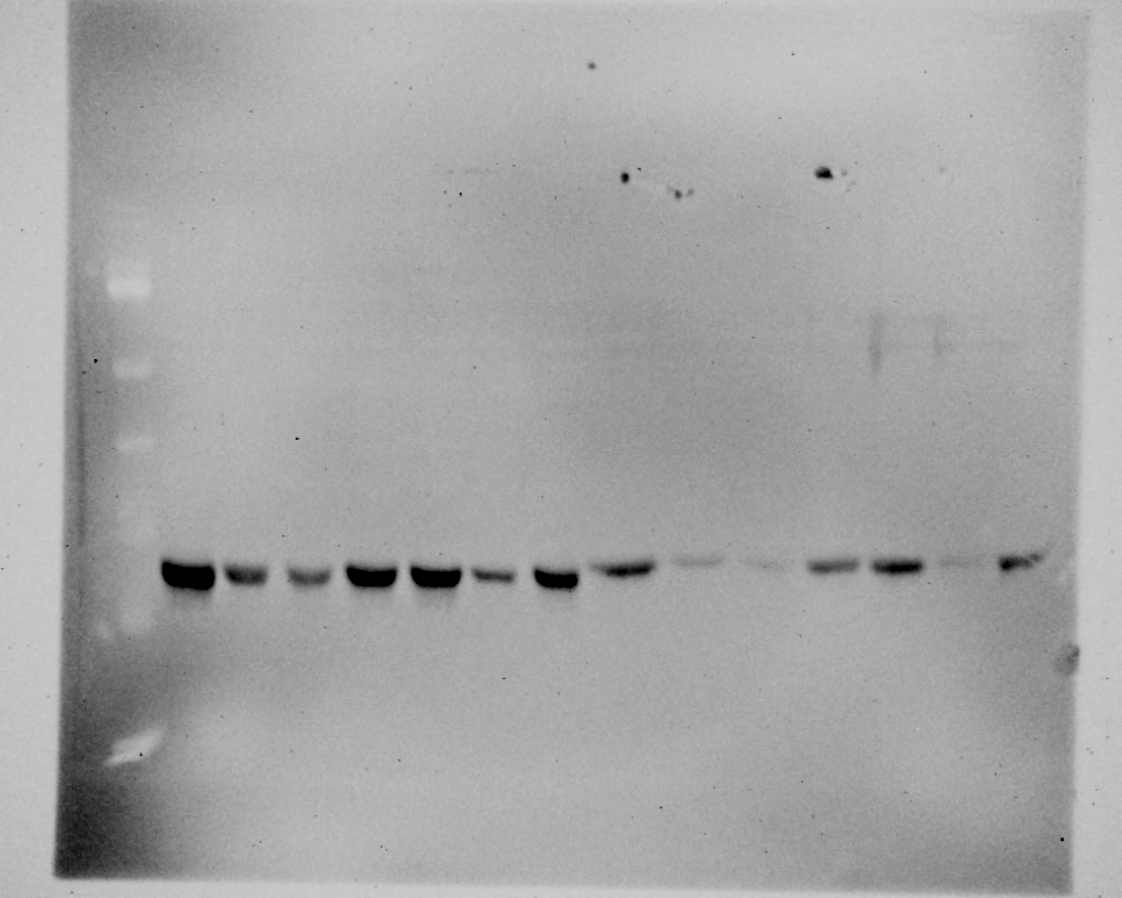

Supplement: Supplementary file 4 — Source Data [file 41467_2023_39909_MOESM4_ESM.zip › SourceData_NCOMMS_23_22106_Lindorff_Larsen/Figure_6/image24.tif]

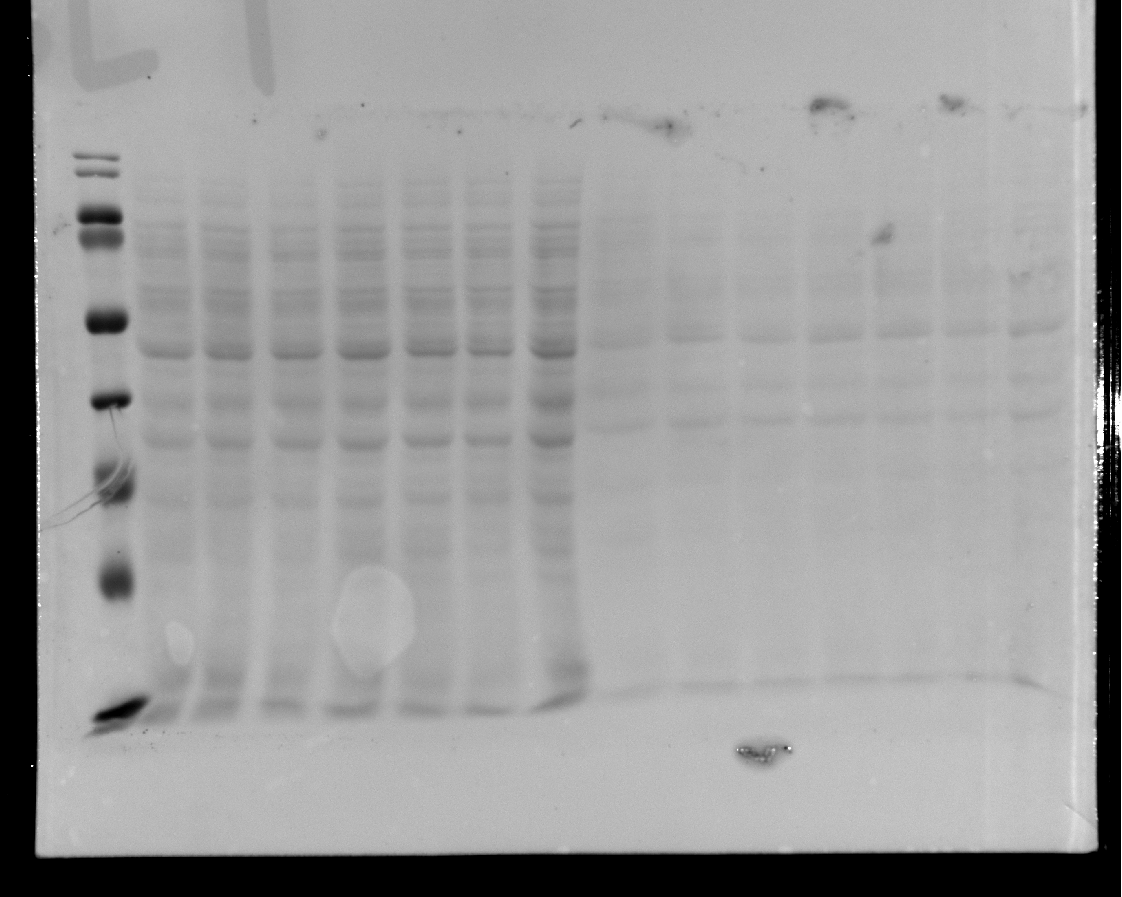

Supplement: Supplementary file 4 — Source Data [file 41467_2023_39909_MOESM4_ESM.zip › SourceData_NCOMMS_23_22106_Lindorff_Larsen/Figure_6/image25.tif]
